# Single-cell profiling of surface glycosphingolipids opens a new dimension for deconvolution of breast cancer intratumoral heterogeneity and phenotypic plasticity

**DOI:** 10.1016/j.jlr.2024.100609

**Published:** 2024-07-30

**Authors:** Jiřina Procházková, Radek Fedr, Barbora Hradilová, Barbora Kvokačková, Josef Slavík, Ondrej Kováč, Miroslav Machala, Pavel Fabian, Jiří Navrátil, Simona Kráčalíková, Monika Levková, Petra Ovesná, Jan Bouchal, Karel Souček

**Affiliations:** 1 Department of Cytokinetics, Institute of Biophysics of the Czech Academy of Sciences, Brno, Czech Republic; 2 International Clinical Research Center, St. Anne's University Hospital Brno, Brno, Czech Republic; 3 Department of Experimental Biology, Faculty of Science, Masaryk University, Brno, Czech Republic; 4 Department of Pharmacology and Toxicology, Veterinary Research Institute, Brno, Czech Republic; 5 Breast Cancer Centre, Masaryk Memorial Cancer Institute, Brno, Czech Republic; 6 Prostate Cancer Centre, Masaryk Memorial Cancer Institute, Brno, Czech Republic; 7 Department of Clinical and Molecular Pathology, Institute of Molecular and Translational Medicine, Faculty of Medicine and Dentistry, Palacký University, Olomouc, Czech Republic; 8 Institute of Biostatistics and Analyses, Faculty of Medicine, Masaryk University, Brno, Czech Republic; 9 Department of Clinical and Molecular Pathology, University Hospital, Olomouc, Czech Republic

**Keywords:** phenotypic plasticity, breast cancer, glycosphingolipids, surface profiling, epithelial cells, stromal-like cells

## Abstract

Glycosylated sphingolipids (GSLs) are a diverse group of cellular lipids typically reported as being rare in normal mammary tissue. In breast cancer (BCa), GSLs have emerged as noteworthy markers associated with breast cancer stem cells, mediators of phenotypic plasticity, and contributors to cancer cell chemoresistance. GSLs are potential surface markers that can uniquely characterize the heterogeneity of the tumor microenvironment, including cancer cell subpopulations and epithelial–mesenchymal plasticity (EMP). In this study, mass spectrometry analyses of the total sphingolipidome in breast epithelial cells and their mesenchymal counterparts revealed increased levels of Gb3 in epithelial cells and significantly elevated GD2 levels in the mesenchymal phenotype. To elucidate if GSL-related epitopes on BCa cell surfaces reflect EMP and cancer status, we developed and rigorously validated a 12-color spectral flow cytometry panel. This panel enables the simultaneous detection of native GSL epitopes (Gb3, SSEA1, SSEA3, SSEA4, and GD2), epithelial–mesenchymal transition markers (EpCAM, TROP2, and CD9), and lineage markers (CD45, CD31, and CD90) at the single-cell level. Next, the established panel was used for the analysis of BCa primary tumors and revealed surface heterogeneity in SSEA1, SSEA3, SSEA4, GD2, and Gb3, indicative of native epitope presence also on non-tumor cells. These findings further highlighted the phenotype-dependent alterations in GSL surface profiles, with differences between epithelial and stromal cells in the tumor. This study provides novel insights into BCa heterogeneity, shedding light on the potential of native GSL-related epitopes as markers for EMP and cancer status in fresh clinical samples. The developed single-cell approach offers promising avenues for further exploration.

## Graphical abstract

**Figure:**
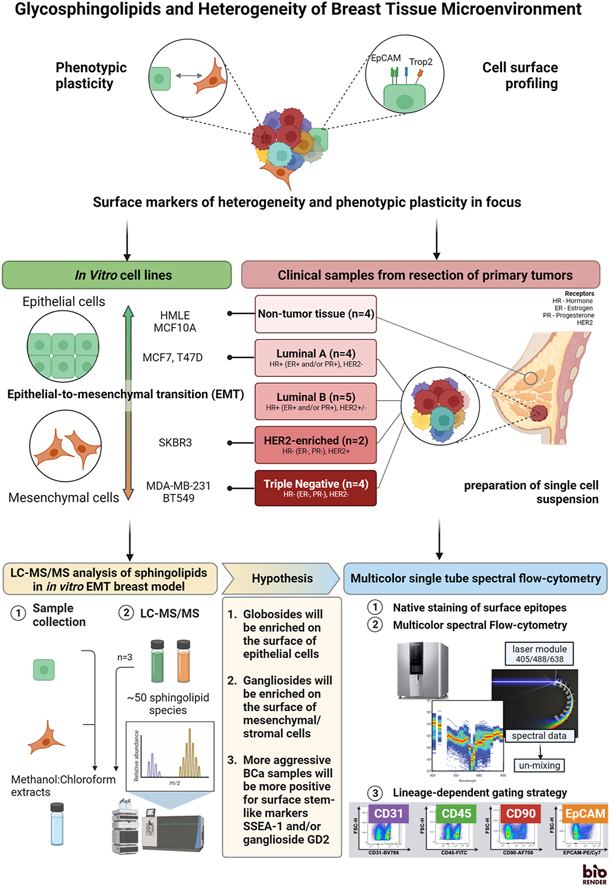

Glycosphingolipids (GSLs) are a class of cellular lipids synthesized primarily in the Golgi apparatus from the sphingolipid ceramide (Cer) through the addition of glucose and subsequently galactose to form lactosylceramide (LacCer; also known as CD17 and CDw17). LacCer is further substituted with sugar molecules of different types and in different orders to form complex GSLs. When compared with normal healthy tissue, studies reporting aberrantly enhanced immunohistochemical staining of GSL-related antibodies in breast cancer tissue make up an essential part of current knowledge. Based on these observations, various GSLs were reported as de novo synthesized or enriched in breast tumors; thus, they became generally considered potent tumor-associated antigens or tumor markers, respectively (1, 2, 3, 4). So far, O-acetyl-GD3, O-acetyl-GT3, N-glycolyl-GM3, SSEA4, globo H, SSEA3, SSEA1, GM2, GD2, and O-acetyl-GD2 are on the list of markers suggested for improved BCa prognosis or development of anti-cancer therapy (5, 6, 7, 8, 9, 10, 11).

Three major series of complex GSL are recognized based on the identity and composition of sugar moieties present in the structure of particular GSL species: ganglio-, globo-, and neo/lacto-series. The complexity of GSLs is further potentiated by diverse fatty acid chains bound to the basic structure of Cer (12). Stage-specific embryonic antigen 1 (SSEA1) (also known as CD15, Lex, or Lewis X antigen) belongs to neolactoseries of GSL species and is a marker of human myeloid cells (e.g., monocytes and granulocytes). It mediates interaction with selectins present on the surface of cells such as endothelial cells (13). Regarding breast tissue, immunohistochemical staining of SSEA1 was not positive in normal breast epithelium, but an antibody recognized this epitope in the acini of the lactating breast and primary tumors (2). SSEA1 correlates with the invasive phenotype in BCa; its enhanced expression in estrogen receptor alpha (ERα)-negative tumors was observed and was also reported as a mediator of adhesion between breast carcinoma cells and activated endothelium (14, 15) This stem cell marker has been observed overexpressed in circulating tumor cells present in the cerebrospinal fluid of breast cancer patients with leptomeningeal metastasis and increased levels of its sialylated form, CD15s, were proposed as circulating tumor biomarker of breast cancer metastasis (16). Globotriaosylceramide (Gb3, also known as CD77), SSEA3, and SSEA4 are GSLs of the globoseries previously reported as present on the surfaces of human peripheral blood cells or embryonic stem cells, respectively (17, 18). Based on the results from the Shiga toxin B subunit overlay method, Gb3 is present in normal breast tissue as well as in breast carcinoma samples (19). Immunohistochemical staining of Gb3 in breast tissue cryosections revealed positive Gb3 signals in vascular endothelial cells and tumor cells (20). Gb3 significantly correlates with ER expression in primary disease and its absence correlates with the increased frequency of lymph node metastases (19). Based on tissue microarray results published by Chang *et al.*, the more glycosylated progeny of Gb3, that is, SSEA3, is not present in normal breast tissue, but 20%–70% of breast cancer cells were reported as positive for this globoside (7). SSEA3 and products of its sialylation (SSEA4) or fucosylation (globoH) are present in breast cancer stem cells and were reported as possible therapeutic targets (6, 11). When investigated from a broader perspective of human solid tumors, an increased positivity of cells for SSEA4 is associated with the loss of an epithelial phenotype and the formation of invadopodia-like migratory structures; thus, SSEA4 has been proposed as a novel marker for a heterogeneous invasive subpopulation of prostate cancer cells (21). GD2 is a disialylated GSL of the ganglioseries and similar to SSEA3, SSEA4, or globo H, its increased presence was detected in breast tumor tissue specimens and on the surface of breast cancer stem cells. Elevated GD2 levels are associated with advanced histological grade and the presence of lymph node metastasis (9, 22, 23).

Generally, GSLs play many biological roles, namely, structural roles, in the maintenance of integrity and polarity of biological membranes and signaling roles as they support the formation of distinct membrane domains, glycosynapses, together with growth factor receptors, tetraspanins, or integrins. They may also serve as receptors for toxins of microbial origin e.g. Shiga-, Vero- or cholera-toxins (24, 25, 26). In particular, GSLs have been reported as potent mediators of phenotypic plasticity during development and cancer progression. They are active molecular players regulating key processes of cancer dissemination i.e. epithelial-to-mesenchymal transition (EMT) and maintenance of stemness (3, 27, 28, 29).

Considering all these facts, we asked the following questions. Do total sphingolipidome and/or GSL-related epitopes present on the surface of breast cancer cells reflect EMP and/or cancer status? How can we deconvolute at the single cell level the degree of surface GSL heterogeneity present in non-tumor and tumor tissue and how can we compare it between analyzed entities? To answer these questions, we combined several approaches. At first, we used mass spectrometry to map sphingolipidome in *in vitro* EMT models and then established and validated a 12-color single-tube flow cytometry protocol compatible with the surface detection of native GSL epitopes (SSEA1, SSEA3, SSEA4, Gb3, GD2) and with the markers of EMT (EpCAM, TROP2, CD9), and in a lineage-specific context (CD45, CD31, CD90) (30, 31). Then, surface profiles of epithelial, mesenchymal, or stromal-like cells were analyzed in *in vitro* breast cell lines representing distinct EMT states and in primary tumors resected from breast cancer patients.

## Materials and Methods

### Cell lines

Epithelial HMLE cells (a kind gift from Prof. R. Weinberg's laboratory, Whitehead Institute for Biomedical Research, MIT) and their mesenchymal counterparts (coded as HMLE-EMT) derived through sequential trypsinization were cultured as described previously (32, 33). Epithelial MCF10A LXSN and mesenchymal MCF10A LXSN V12 cells overexpressing the mutant form of Ras G12V were a kind gift from Prof. Ben Ho Park (Johns Hopkins University) and were handled as described previously (33). T47D, SKBR3, MCF7, and MDA-MB-231 were purchased from American Tissue Culture Collection (ATCC) and cultured according to the provider's recommendations; T47D in RPMI 1640 medium (Gibco, ThermoFisher Scientific (TFS)) with 10% of fetal bovine serum (FBS; TFS), SKBR3 in McCoy's medium (TFS) with 10% FBS, MCF7 in MEM with 10% FBS and MDA-MB-231 in RPMI 1640 medium with 10% FBS. All cell lines were cultured in the presence of 1% Penicillin/Streptomycin (Biosera) at 37°C and 5% CO_2_, passaged twice a week, routinely checked for mycoplasma contamination with PCR, authenticated using AmpFLSTR Identifiler Plus PCR Amplification Kit (TFS) to verify their origin and used for experiments within 10 passages.

### Analysis of sphingolipid species by HPLC-MS/MS

Sample preparation and analysis of sphingolipid (SL) and GSL species was performed as described previously (34). HMLE and HMLE-EMT cells were cultured in technical triplicates, counted, and used for the preparation of methanol extracts. Data were normalized based on the number of cells per sample and the amount of total protein per sample. Samples from three independent biological repetitions were analyzed each in triplicate. SL species were separated by reversed-phase HPLC (Dionex Ultimate 3000, Thermo Scientific) on Gemini column C18, 5 μm, 250 × 4,6 mm (Phenomenex); glucosylceramide (GlcCer), galactosylceramide (GalCer) were each separated through normal-phase HPLC using a Spherisorb column 5 μm Silica, 2,1 × 250 mm (Waters Corporation, Ireland); gangliosides and globoside Gb3 were separated by reverse phase HPLC on a ZORBAX Eclipse C8 4,6 × 250 mm column (Agilent Technologies). A tandem mass spectrometer (Hybrid triplequad QTRAP 4500, AB Sciex) was operated under the following conditions: ESI in positive mode, drying air, collision energy, and fragmentor voltage were optimized for each species, with the spectrometer running in multiple-reaction monitoring scan mode. Mass spectrometry standards used for the quantification of each assessed lipid species were purchased from Avanti Polar Lipids (Alabaster). The content of individual sphingolipid species was calculated from the average chromatographic peak area and expressed as the amount of appropriate internal standard. Total normalized levels of sphingolipid species analyzed in all repetitions are summarized in supplemental Table S1.

### RT-qPCR

For the isolation of total RNA from HMLE and HMLE-EMT cells, a High Pure RNA Isolation kit (Roche) was used following the manufacturer’s instructions. One microgram of total RNA was reversely transcribed into cDNA using a High-Capacity RNA-to-cDNA Kit (Life Technologies, TFS) using both types of primers: random hexamers and poly(T) primers. Quantitative PCR was performed as described previously using an LC Probe Master Kit (Life Technologies, TFS) and TaqMan assays specified in supplemental Table S2 (35). The recommended conditions for PCR reaction: initial denaturation step at 95°C for 5 min, 45 cycles at 95°C for 10 s, 60°C for 30 s, and 72°C for 1 s with fluorescence acquisition. Relative mRNA levels were calculated and visualized as 2^-ΔCt^. A *GAPDH* TaqMan assay was used as a housekeeping gene.

### Western blotting

Total protein extracts were prepared from tested breast cell lines and processed for western blotting as described previously using 1% SDS lysis buffer (1% SDS, 100 mM Tris-HCl, pH 7.4) (33). Antibodies used for immunodetection are summarized in supplemental Table S2. Uncropped western blots from all three independent replicates are presented in supplemental Figs. S6 and S7.

### Breast cancer patient samples

All clinical samples were sourced from Masaryk Memorial Cancer Institute (MMCI), and the study was approved by the Ethical Committee of the MMCI (Ref. No. 2017/1894/MOU). The experiments were conducted with the understanding and written consent of each patient and in accordance with the principles of the Declaration of Helsinki. On the day of surgery, fresh breast cancer tissues from patients were evaluated by a certified pathologist and stored for 24 h in MACS® Tissue Storage Solution (Miltenyi Biotec). The description of the patient cohort (a total of 18) is provided in detail in supplemental Table S4.

### Preparation of single-cell suspensions from breast tissues

Tissue samples were processed as described with several modifications (31). The enzymatic digestion of tissue with dispase II and collagenase type I was optimized for 6 h at 37 °C using 15 rpm agitation. After treatment of samples with DNase I (Roche), incubation in ACK lysis buffer (155 mM ammonium chloride, 10 mM potassium bicarbonate, and 100 μM EDTA solution in sterile MQ water) to lyse the red blood cells, filtration through a 70 μm strainer and wash with PBS, the number of cells in the cellular suspension was calculated using a CASY Cell Counter (Roche) and up to 1 million cells per staining reaction were used for the native staining of cellular surfaces and subsequent analysis through spectral flow cytometry.

### Multi-color staining and analysis of cell surfaces by spectral flow cytometry

Up to 1 million cells (cell lines or clinical samples) were stained in PBS for viability for 20 min at 4 °C and washed with ice-cold PBS and incubated for 20 min at 4 °C in the dark with a panel of antibodies recognizing specific epitopes or with their respective isotype controls, each prepared in 100 μl of staining buffer (1% BSA, 0.1% NaN_3_ in PBS) supplemented with Super Bright Complete Staining Buffer (eBioscience, TFS). After the final wash step, cells were resuspended in 200 μl of staining buffer and analyzed using Spectral Cell Analyzer SONY SP6800 (Sony Biotechnology). Panels with antibodies and isotype controls are listed in supplemental Table S3. Compensated FCS files were exported and analyzed using the FlowJo software (v10.8.0, BD). Only viable (Zombie NIR-negative) single cells (FSC-H/FSC-A) with excluded debris (SSC-A/FSC-A) were further analyzed. The Multidimensional reduction algorithm Fit-SNE implemented in the FlowJo software was used to produce 2D maps (tSNE2/tSNE1) (36). The color-coding of events visualized in maps stands for the relative intensity of selected markers from the panel (highest intensity in red, lowest in blue). FlowSOM analysis, an unsupervised technique for clustering and dimensionality reduction, was used to enable the identification and self-organizing visualization of clusters in tSNE dimensions (37).

Manually pre-gated flow cytometric data from in vitro and clinical samples measured on a spectral analyzer were processed in RStudio (v. 2023.02.0, Posit Software, PBC), and data were centered and scaled. Correlations of parameters together with statistics and correlograms with complete hierarchical clustering were produced using the “corrplot” package (38, 39). Only significant parameter correlations (r > 0.25 or r < −0.25, *P* > 0.05) are shown in the plots. Heatmaps were generated from median fluorescence index (MFI) values identified in each cluster using the FlowSOM algorithm and visualized using the Morpheus tool (https://software.broadinstitute.org/morpheus).

### Statistical analysis

Statistical analyses and chart visualizations were performed using the R software (version 4.1.1, R Core Team, 2021) and the GraphPad Prism software (version 9.0.2, GraphPad Software). Detailed information describing statistical analyses used for the evaluation of each dataset is presented in the figure legends.

## Results

### Metabolic profiling of epithelial-mesenchymal plasticity in breast in vitro models revealed a shift in GSL levels

**Fig. 1:**
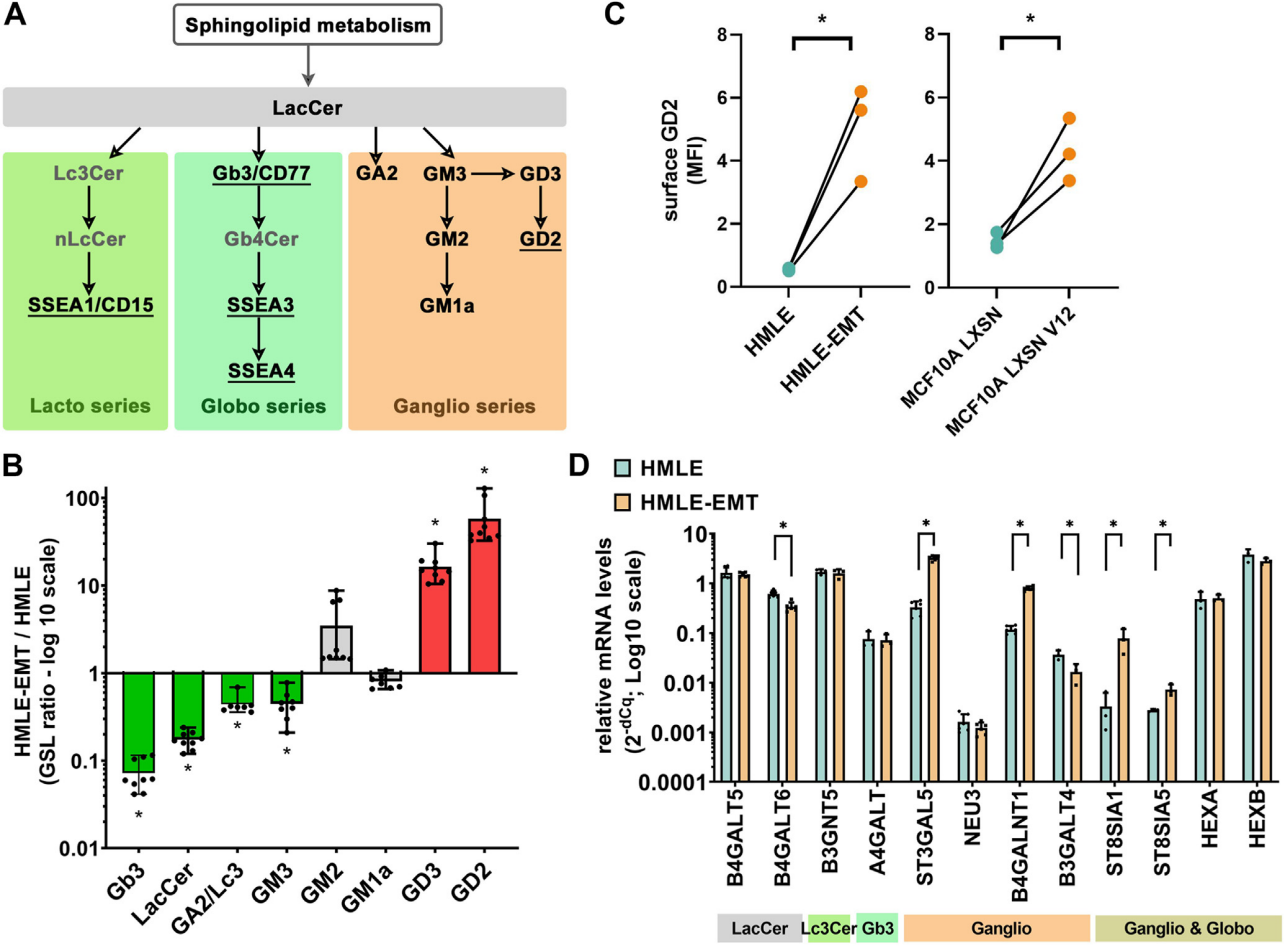
Alterations of sphingolipid metabolism in in vitro models of breast EMT. A: The metabolic origin of glycosylated sphingolipids investigated in this study is summarized in a simplified scheme. GSL species analyzed for surface expression are underlined. Species in grey color were not analyzed by HPLC-MS/MS. B: Normalized levels of GSL species are shown in supplemental Fig. S1A and herein visualized as the ratio between HMLE and HMLE-EMT cells. GSL species significantly enriched in HMLE cells are shown in green, and those significantly enriched in HMLE-EMT cells in red and gray color depict no statistical significance (three independent repetitions done in technical triplicate, ∗*P* < 0.01, paired *t* test). C: Surface presence of native epitope recognized by GD2 antibody was analyzed by flow cytometry in in vitro breast EMT models, i.e. HMLE/EMT and MCF10A LXSN/V12 (n = 3, ∗*P* < 0.01; unpaired *t* test). D: Relative mRNA levels of genes encoding enzymes of GSL metabolism were measured in HMLE and HMLE-EMT cells; genes significantly upregulated or downregulated in mesenchymal cells are depicted by an asterisk (three independent repetitions in technical duplicate, *P* < 0.05; unpaired *t* test).

To directly address the metabolism of sphingolipids (SLs) and GSLs (Fig. 1A) in the context of breast epithelial-mesenchymal plasticity, we took advantage of a well-established model of in vitro EMT pair, epithelial HMLE cells and their mesenchymal counterparts (HMLE-EMT) (32, 33). High-performance liquid chromatography combined with tandem mass spectrometry (HPLC-MS/MS) revealed significant alterations in the profiles of GSL species between these cell lines. All analyzed lipid species and their normalized total levels detected in triplicates in three independent repetitions are summarized in supplemental Table S1. Total levels of species of dihydrosphingomyelin (dhSM), lactosylceramide (LacCer), Gb3, GD3, and GD2 significantly differed between epithelial and mesenchymal cells (supplemental Fig. S1A). In particular, the analysis revealed profound changes in the GSL ratio between HMLE-EMT and HMLE cells, pointing out the globoside Gb3 as a GSL enriched in epithelial cells and the ganglioside GD2 as a GSL enriched in mesenchymal cells (Fig. 1B). The analysis also showed that neither of the detected GSL species was absent in any of the tested samples, thus indicating the overall presence of GSL species in both phenotypes and the existence of a shift in the GSL synthesis alongside the EMT trajectory.

To validate the increased presence of GD2 in mesenchymal cells, especially on the cell surface, single-cell detection of the native GD2 epitope in epithelial and mesenchymal cells was performed using flow cytometry (FC). For this purpose, pairs of epithelial (HMLE, MCF10A LXSN) and mesenchymal (HMLE-EMT, MCF10A LXSN V12) breast cell lines were stained with a fluorescently conjugated GD2-specific antibody and analyzed in viable cells. A significant increase in median fluorescence index (MFI) and cellular positivity for GD2 was confirmed in both mesenchymal cell lines (Fig. 1C and supplemental Fig. S1B, C).

Taken together, while the HPLC-MS/MS method informs about the profile of glyco/sphingolipids present in the entire mass of cells, here we employed flow cytometry to get information about the presence/absence of native GSLs on the cellular surface. The mechanisms regulating the final presence of particular GSLs on the surface are unclear and may underlie the observed discrepancies when comparing these two methods. Nevertheless, GD2 and Gb3 detected on the cell surface through FC were also detected in tested cell lines through HPLC-MS/MS.

### Expression levels of genes driving the synthesis of gangliosides correspond with the metabolic shift observed in mesenchymal cells

To map transcriptional changes in the landscape of GSL metabolism, a list of genes encoding relevant enzymes was compiled and their relative mRNA levels were analyzed. Reduced mRNA levels of LacCer synthase encoded by gene *B4GALT6* corresponded with lower concentrations of LacCer, a precursor of complex GSL synthesis; however, we can also expect that a significant part of the LacCer pool was used for enhanced consecutive ganglioside synthesis. Expression profiles of genes (*ST3GAL5*, *B4GALNT1*, *ST8SIA1*, and *ST8SIA5*) involved mainly in the synthesis of GD3 and GD2 from their direct (GM3, GD3) or distant precursors (LacCer) were significantly upregulated in mesenchymal cells (Fig. 1D). Meanwhile, the expression levels of genes encoding enzymes converting LacCer into GSLs of the globoseries (*A4GALT*) or neo/lactoseries (*B3GNT5*) were not significantly altered similarly to genes driving catabolic reactions in the metabolism of the globo- and/or ganglioseries (*NEU3*, *HEXA*, *HEXB*). In conclusion, expression data were in accordance with the enhanced synthesis of the gangliosides GD3 and especially GD2 in mesenchymal cells and did not indicate simultaneous transcriptional deregulation of globo- or neo/lacto-GSL synthesis.

### Interpretation of EMT status based on surface profiles of GSLs is cell model-dependent

**Fig. 2:**
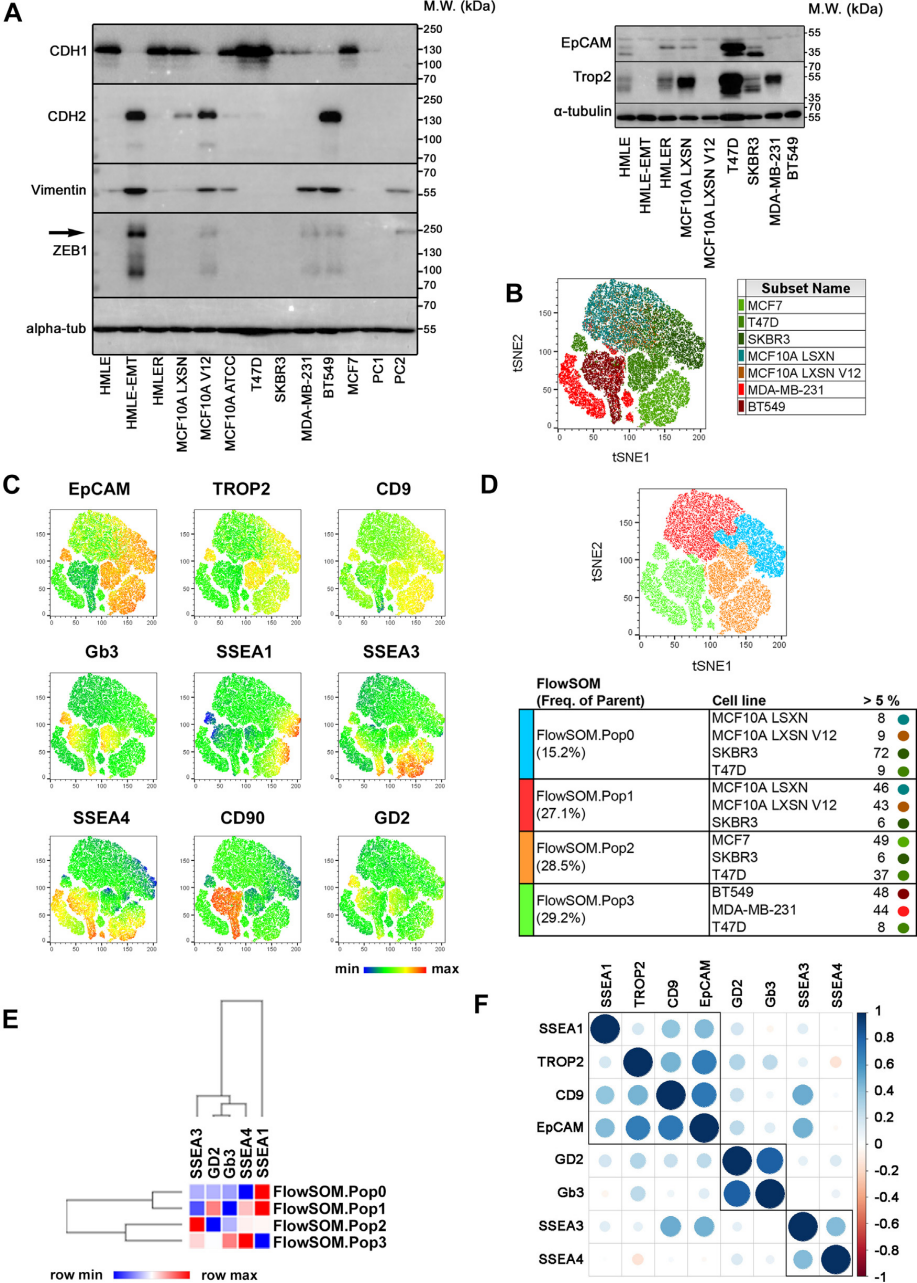
Surface profiling of breast in vitro epithelial and mesenchymal cell lines. A: Protein levels of EMT markers were detected in total lysates harvested from the panel of breast cell lines. The figure represents 1 of 3 biological repetitions; uncropped western blots from all 3 repetitions are shown in supplemental Figs. S6 and S7. The arrow depicts a specific band for ZEB1 protein, and molecular weight (M.W.) is shown for each protein in kilodaltons (kDa). PC1 and PC2 stand for positive controls. B–F: Cell lines were stained with a multi-color panel of antibodies specified in supplemental Table S3 and analyzed for the presence of surface GSL-related epitopes and EMT markers. Concatenate of 15.000 cells per sample preceded to two-dimensional reduction analysis performed by FitSNE algorithm to show heterogeneity in surface expression of analyzed molecules in depicted cell lines. B: tSNE map of pooled sample colored by (C) expression of EMT markers and GSLs or by (D) identified FlowSOM clusters. The table shows the percentual distribution of samples in individual FlowSOM clusters. E: Heatmap depicts hierarchical clustering and expression intensity of GSLs in identified FlowSOM clusters. F: Correlation matrix of analyzed epitopes as calculated from single cell measurements of fluorescent intensities.

Considering the EMT-dependent alterations in GSL metabolism and the existence of surface fingerprints specific for epithelial and mesenchymal cells (30), the one-tube protocol for the surface profiling of GSLs together with protein EMT markers was established to test the EMT-dependent surface distribution of GSLs in breast in vitro models of different EMT statuses. At first, cell lines were screened for the expression of EMT-related protein markers (CDH1, CDH2, VIM, ZEB1, EpCAM, TROP2; Fig. 2A) to confirm the epithelial status of T47D, SKBR3, MCF7, HMLE and MCF10A LXSN, the mesenchymal status of HMLE-EMT, MCF10A LXSN V12 and BT549, and the hybrid status of MDA-MB-231. A panel of fluorochrome-conjugated primary antibodies recognizing epitopes of SSEA1/CD15, SSEA3, SSEA4, GD2, and Gb3/CD77 was then introduced and merged with the panel of antibodies recognizing surface EMT markers EpCAM/CD326, TROP2, and CD9 and the lineage-specific markers CD45, CD31, and CD90/Thy1 (supplemental Table S3) (30, 31). Paired cell suspensions were also incubated with the isotype controls corresponding to antigen-specific antibodies and the percentage of positivity was determined based on the gating strategy excluding the cells negative for the inspected marker. Finally, the surface expression of GSL-related epitopes between phenotypic groups (five epithelial vs. four mesenchymal cell lines) was compared. supplemental Fig. S2 summarizes MFI values and the percentage of positivity for every analyzed surface molecule in all inspected cell lines. Analysis revealed the general absence of surface GSLs (except for GD2) in HMLE and MCF10A LXSN EMT models. Due to the positivity of both epithelial and mesenchymal HMLE cell lines for the CD90 surface mesenchymal marker (bipotential precursor characteristics of HMLE model) (40), they were excluded from further analysis. Therefore, only flow cytometric data from T47D, SKBR3, MCF7, MDA-MB-231, BT549, and MCF10A LXSN/V12 cells were further analyzed through automated dimensionality reduction algorithm fast Fourier transform-accelerated interpolation-based t-distributed stochastic neighbor embedding (Flt-tSNE). The tSNE map in Fig. 2B visualizes a pool of all samples colored in Fig. 2C through the expression of EMT and GSL epitopes and in Fig. 2D through FlowSOM clusters. tSNE and FlowSOM analyses were calculated from GSL-related fluorescent signals only to test whether surface GSL epitopes alone can discriminate between epithelial and mesenchymal phenotypes.

FlowSOM analysis identified four distinct clusters, assigned as Pop0, Pop1, Pop2, and Pop3, with Pop2 being formed by only epithelial cells and cluster Pop3 mainly by mesenchymal cells (see table in Fig. 2D). Considering Pop2 and Pop3 FlowSOM clusters as stratifying in terms of EMT phenotype, surface expression of Gb3, GD2, SSEA3, and SSEA4 was present in both of them, while SSEA1 expression was detected only in epithelial cluster Pop2 (Fig. 2C, D). As expected from our previous study (30), the expression patterns of TROP2, EPCAM, and CD9 overlapped and mutually correlated in epithelial cells (Fig. 2C, F). CD90+ mesenchymal BT549 cells formed an autonomic tSNE region that was also positive for GSLs except for SSEA1 (Fig. 2C, D). The heatmap shown in Fig. 2E compares GSL expression in all identified FlowSOM clusters with SSEA1 and SSEA3 being more prominently expressed in the pro-epithelial FlowSOM cluster Pop2 and GD2, Gb3, and SSEA4 being more prominently expressed in the pro-mesenchymal cluster Pop3. Finally, while correlation analysis confirmed the association of SSEA1 expression together with the epithelial markers EPCAM, TROP2, and CD9 (Fig. 2F), it did not confirm the same expectations for Gb3. Moreover, contrary to HPLC-MS/MS-based assumptions, surface Gb3 positively correlated and clustered mainly with GD2 (Fig. 2C, F).

In conclusion, when compared with metabolic profiling, the surface analysis of GSL-related epitopes appears to distinguish epithelial and mesenchymal phenotypes only in in vitro EMT models of the same origin (HMLE vs. HMLE-EMT, MCF10A LXSN vs. MCF10A LXSN V12), whereas other cell lines show a specific model-dependent GSL pattern. The expression of Gb3, GD2, SSEA3, and SSEA4 was detected in both phenotypic entities, while SSEA1 was present on the surface of epithelial cells. Meanwhile, FlowSOM analysis helped to visualize the preferential presence of SSEA3 on the surface of epithelial-like cells (Pop2), while Gb3, GD2, and SSEA4 were more prominently expressed on the surface of the mesenchymal-like cells (Pop3) (Fig. 2E).

### Native GSL-related epitopes are present on the surface of both tumor and non-tumor cells isolated from patient breast tissues

**Fig. 3:**
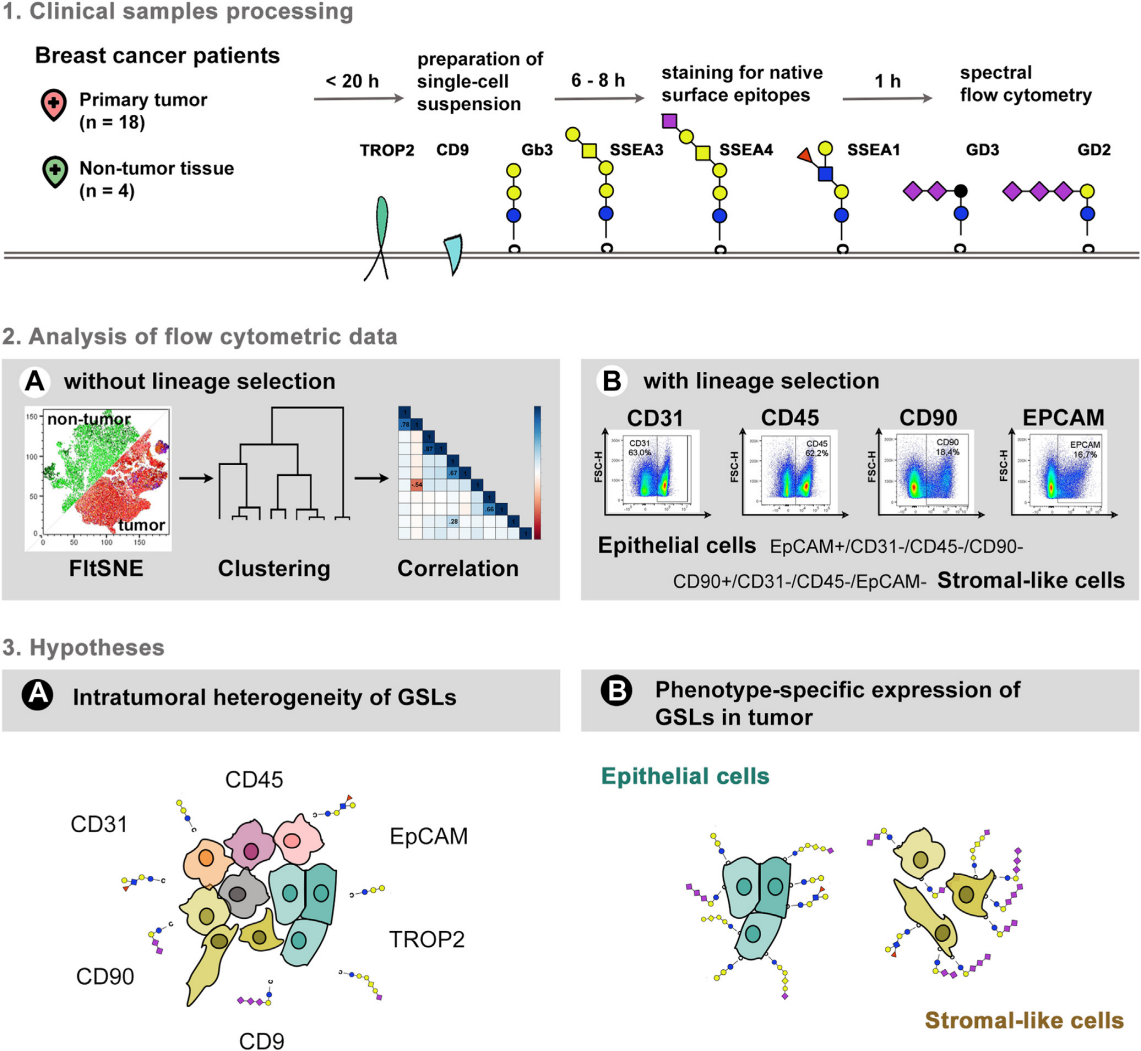
The strategy of clinical samples surface profiling. Breast tissue samples were processed according to the standard experimental procedure summarized in panel 1 and described in the methods. Single-cell suspensions were stained with a panel of antibodies specified in supplemental Table S3 and analyzed without lineage selection for intratumoral heterogeneity (panel 2A) or with lineage selection for phenotypical plasticity (panel 2B) to address hypotheses summarized in panels 3A and 3B, respectively.

In the context of cancer progression, EMT is a critical process, and GD2 is a validated target for high-risk neuroblastoma treatment (41). GSLs are implicated in ovarian and breast cancer (6, 28). Therefore, we next examined GSL-related surface expression in primary breast tumors from patients without a diagnosed metastatic disease, along with limited samples of non-tumor tissue. Primary breast tumor is composed of cancer cells and a variety of stromal cells, including endothelial cells, fibroblasts, immune system cells, or mesenchymal stem cells. In this study, we aimed to analyze the intratumoral heterogeneity of native surface GSLs, compare it with paired non-tumor counterparts if possible, and put it in the context of the epithelial-mesenchymal plasticity of primary tumors. To achieve this, we established a one-tube multi-color flow cytometry protocol for the native staining of GSL-related epitopes in conjunction with lineage- and phenotype-specific markers, namely CD45 (hematopoietic cells), CD31 (endothelial cells, leukocytes, and thrombocytes), CD90 (stromal, mesenchymal cells), and EpCAM, TROP2, and CD9 (epithelial cells). Based on lineage markers, we successfully identified and further analyzed the epithelial-like (EpCAM+/CD45-/CD31-/CD90-) and stromal-like (CD90+/CD45-/CD31-/EpCAM-) subpopulations present within the breast tissue microenvironment. The strategy of clinical sample processing and analysis is illustrated in Fig. 3, including the gating strategy and tested hypotheses. For the tSNE-based multidimensional reduction and FlowSOM clustering fluorescent signals of all analyzed epitopes [i.e. lineage markers (CD45, CD31, and CD90), phenotype markers (EpCAM, TROP2, and CD9) and GSLs (SSEA1, SSEA3, SSEA4, Gb3, GD2)] were used because our goal was to map surface GSLs together with the most frequent cell types (epithelial, stromal, hematopoietic, endothelial) present in clinical samples.

Four non-tumor samples (NT) were obtained from three patients, one diagnosed with the luminal B BCa subtype and two with triple-negative breast cancer (TNBC) (supplemental Table S4). Based on previously reported immunohistochemical studies showing a general absence of GSLs in healthy mammary tissue, we expected a low surface presence of native GSL-related epitopes in NT samples. The overall surface presence of most GSLs was low except for that of GD2 (supplemental Fig. S3A, C). Moreover, the MFI values of the epithelial surface markers EpCAM and TROP2 were also very low in NT samples when compared to tumor samples (BCa). However, the non-parametric Mann-Whitney test with the correction for multiple testing did not reveal statistical differences between non-tumor and tumor samples, mainly because of a disproportionate number of samples in the non-tumor (n = 4) and tumor groups (n = 18) (supplemental Fig. S3A).

Five FlowSOM clusters (FlowSOM.Pop0-4) were identified and equally distributed across NT samples (supplemental Fig. S3B). The FlowSOM.Pop0 and Pop2 clusters harbored cells with high expression levels of CD45, CD31, and CD9 and differed in the expression levels of CD90, GD2, Gb3, and SSEA3. These clusters represent subpopulations of hematopoietic and endothelial cells. They were characterized by a strong positive correlation between GD2, Gb3, and SSEA3 in cluster Pop0, and between SSEA4 and EpCAM or TROP2 in cluster Pop2 (supplemental Fig. S4A). Cluster Pop4 was characterized mainly by the high expression level of CD90 together with CD9 and likely represents a subpopulation of stromal-like cells. The remaining clusters Pop1 and Pop3 were characterized by low expression levels of CD31 and CD45 and differed in the expression levels of CD90, CD9, SSEA3, SSEA4, and GD2 (supplemental Fig. S3C, D). As mentioned above, the surface expression level of EpCAM and TROP2 was generally low in non-tumor tissue; this fact is reflected in tSNE projections colored by their expression and in the vastly increased percentual frequency of the stromal-like cells (CD90+/CD31-/CD45-/EpCAM-) in comparison to epithelial cells (EpCAM+/CD45-/CD31-/CD90-) (supplemental Figs. S3C, D and S4C). Therefore, an epithelial-like cluster enriched for EpCAM and TROP2 expression and simultaneously characterized by the low expression levels of CD45, CD31, and CD90 was not identified by FlowSOM analysis in NT samples. tSNE projections colored by EpCAM and TROP2 expression visualize a very small region positive for both markers in the FlowSOM.Pop3 cluster. This region was also characterized by higher expression levels of SSEA1, SSEA3, and SSEA4 (supplemental Fig. S3B–D). Further analysis confirmed a strong positive correlation between EpCAM and TROP2 in the FlowSOM.Pop3 cluster (supplemental Fig. S3E). Interestingly, a correlation analysis also revealed a strong (r ∼ 0.5) positive association between TROP2, SSEA1, CD45, and CD31 in the stromal-like compartment (FlowSOM.Pop4, supplemental Fig. S3E). Taken together, five tissue subpopulations were identified in NT samples, with surface GSLs being heterogeneously expressed mainly in three of them but in very low amounts except for GD2.

**Fig. 4:**
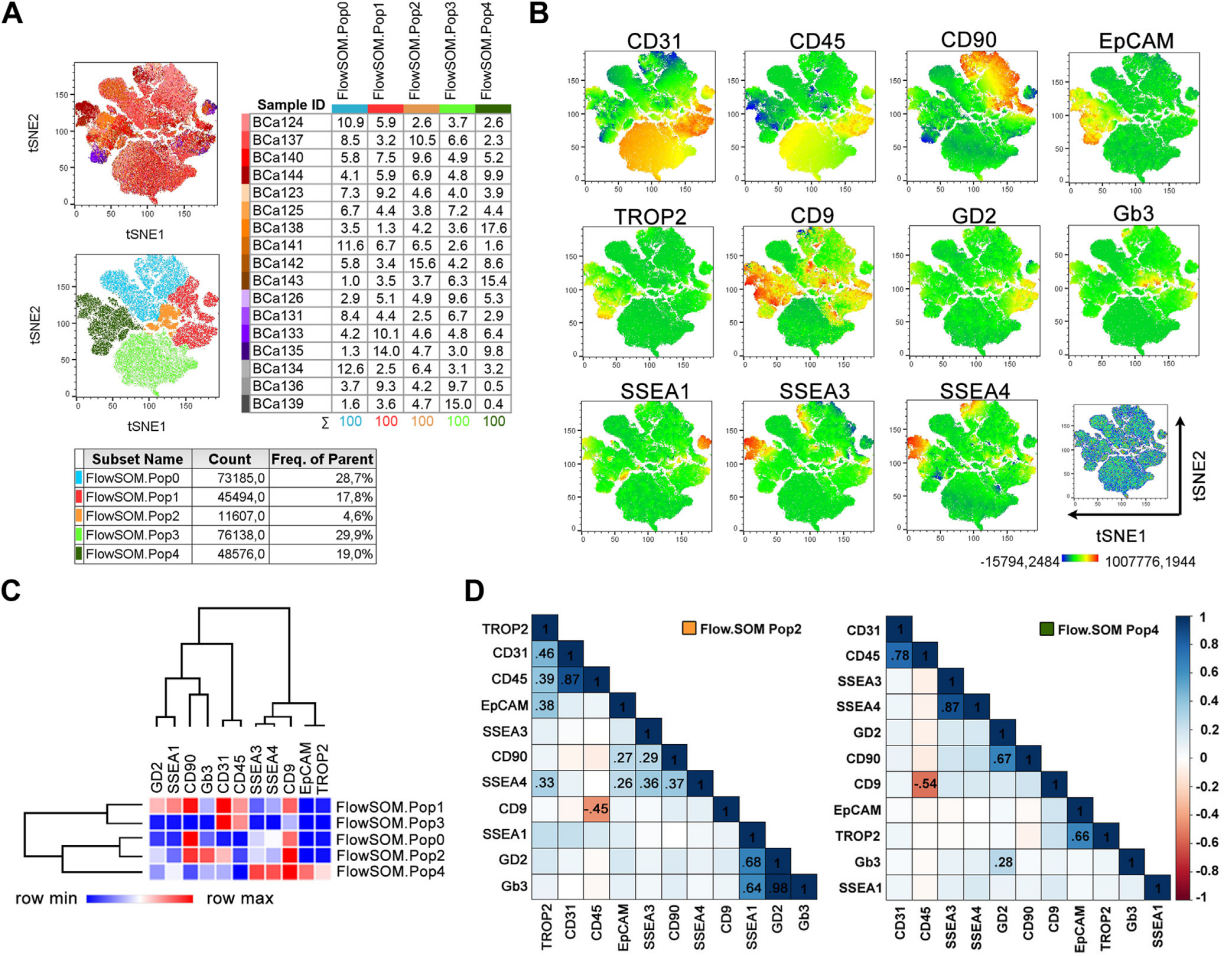
Surface profiling of GSL-related epitopes in breast tumors. Single suspensions isolated from primary breast tumors were stained with a multi-color panel of antibodies specified in supplemental Table S3 and analyzed for the presence of surface GSL-related epitopes and EMT markers. The pool of cells (15.000 per sample) from 17 patients was reduced by the FitSNE algorithm to show intratumoral heterogeneity in the expression of surface molecules. A: tSNE maps are colored by Sample ID (up) or by FlowSOM clusters (down). Tables show the percentual distribution of samples in individual FlowSOM clusters (up) or distribution of FlowSOM clusters (down) in pooled cells. B: tSNE maps colored by surface expression of analyzed epitopes. C: Heatmap depicts hierarchical clustering and expression intensity of GSLs in identified FlowSOM clusters. D: FlowSOM cluster-specific correlation matrices of analyzed epitopes as calculated from single-cell fluorescence intensities. Only statistically significant correlation coefficients r > 0.25 (blue) or r < −0.25 (red) are shown.

In primary breast tumors, FlowSOM analysis identified five clusters (FlowSOM.Pop0-Pop4) based on the surface expression of GSL, EMT, and lineage markers, and their distribution was again detected across all tested samples (Fig. 4A). Hematopoietic and endothelial subpopulations defined by high CD31 and CD45 expression levels were represented mainly by two distinct clusters, FlowSOM.Pop1 and Pop3, differed from each other by the increased expression levels of GD2, Gb3, SSEA1, CD90, SSEA4, and CD9 in the FlowSOM.Pop1 cluster (Fig. 4B, C). The single-cell analysis confirmed a positive correlation of hematopoietic and endothelial subpopulations with EpCAM, TROP2, and SSEA3 in FlowSOM.Pop1 (supplemental Fig. 4A). When compared, non-tumor FlowSOM.Pop0 and Pop2 clusters harbor similar types of cells (likely hematopoietic and endothelial) to those detected in the tumor FlowSOM.Pop3 and Pop1 clusters, respectively, except for the SSEA3 and SSEA1 expression patterns being reciprocally inverted between the NT and tumor samples (supplemental Fig. S3C and Fig. 4C). Finally, based on hierarchical clustering and marker expression visualized in tSNE projections, epithelial and stromal-like subpopulations of tumor tissues were represented by the FlowSOM clusters Pop4 and Pop2 and Pop0, respectively. A strong positive correlation between EpCAM and TROP2, GD2 and Gb3 was revealed in various clusters identified in both tissue samples. Interestingly, the surface expression of therapeutic target TROP2 strongly correlated (r ≥ 0.7) with the surface expression of EpCAM in epithelial-like subpopulation and with SSEA4 in non-tumor hematopoietic/endothelial (FlowSOM.Pop2) or tumor stromal-like (FlowSOM.Pop0) subpopulations both enriched for CD90 and CD9 expression (supplemental Figs. S3D, E and S4A). Similarly, the expression of GD2, a reported surface marker of breast cancer stem cells, strongly (r ∼ 0.7) correlated with the mesenchymal marker CD90 and only on the surface of tumor cells (FlowSOM.Pop4, Fig. 4D). Finally, SSEA3 and SSEA4 strongly correlated with each other in the epithelial-like subpopulation identified in tumor samples (r ∼ 0.9; FlowSOM.Pop4, Fig. 4D).

### Surface expression of GSL-related epitopes significantly differs between epithelial and stromal-like cells present in breast cancer tissue

**Fig. 5:**
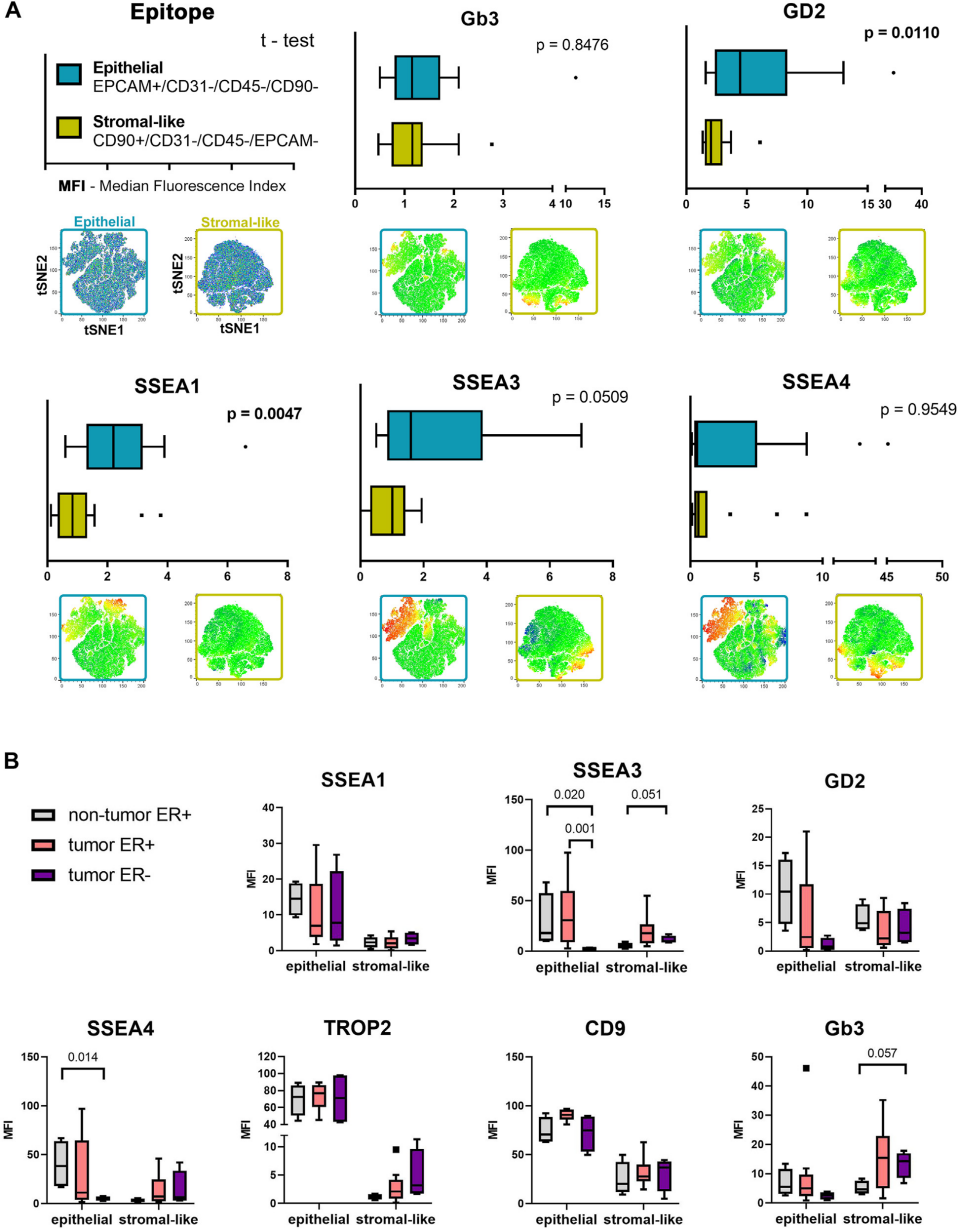
Surface profiling of GSL-related epitopes in epithelial and stromal-like tumor cells. A: Median fluorescence indexes (MFI) were calculated for each GSL-related epitope detected on the surface of epithelial or stromal-like cells (bar charts). tSNE maps located below bar charts visualize the surface expression pattern of each GSL in either epithelial cancer cells (blue frame) or in stromal-like cancer cells (yellow frame). Data were evaluated by unpaired *t* test; the *P*-value describing the difference in epitope expression between epithelial and stromal-like cancer cells is shown in the upper corner of each bar chart and highlighted in bold when significant, *P* < 0.05. B: Tukey plots show MFI values of surface GSLs or EMT markers in epithelial or stromal-like cells detected in non-tumor or tumor samples stratified based on ER status. Data were evaluated for statistical significance by ANOVA test with Holm post-hoc *P* value correction; *P* values ∼0.05 are shown.

Based on in vitro results shown in Fig. 2, we expected high TROP2, CD9, and SSEA1 positivity in epithelial cancer cells and the heterogeneous positivity of both epithelial and stromal-like cancer cells for SSEA3, SSEA4, GD2, and Gb3. When compared with stromal-like cells (CD90+/CD31-/CD45-/EpCAM-), the epithelial subpopulation defined as EpCAM+/CD31-/CD45-/CD90-harbored a higher percentage of SSEA1-positive, TROP2-positive, and CD9-positive cells (Fig. 5 and supplemental Fig. S4B, C). CD9 expression was detected in all clusters identified by FlowSOM (Fig. 4). SSEA3 and SSEA4 were present preferentially on the surface of EPCAM + cells, however, the stromal-like subpopulation, which was enriched in the FlowSOM.Pop0 cluster and represented by CD90+/CD31-/CD45-/EpCAM-cells, also displayed mild positivity, which strongly increased in the case of SSEA3 in TNBC samples (Fig. 4 and supplemental Fig. S4B, C). When the epithelial and stromal-like subpopulations were compared for the positivity of Gb3 and GD2 independently of the BCa subtype, we observed a significant enrichment of epithelial cells only for GD2 positivity (Fig. 5A). Importantly, a trend of increased positivity for GD2, Gb3, and SSEA3 was observed in the stromal-like subpopulation of the TNBC microenvironment (supplemental Fig. S4C).

To analyze the impact of ER status on the presence of native GSL-related epitopes, clinical samples were divided into two groups: ER-positive tumors (n = 11) and ER-negative tumors (n = 5). supplemental Fig. S5 shows the similar overall positivity of both tumor groups for TROP2, EpCAM, CD9, GD2, and Gb3 and highlights interesting alterations in cellular positivity for SSEA1, SSEA3, or SSEA4 between tested groups. In the subsequent analysis, a comparative evaluation was conducted between epithelial and stromal-like cells concerning the expression of GSLs, TROP2, and CD9, contingent upon the ER status. It was observed that the fluorescence intensity of GD2, SSEA3, SSEA4, and Gb3 exhibited a notable decline as the ER status shifted towards negativity in epithelial cells (Fig. 5B). Simultaneously, the stromal-like subpopulation defined in ER-negative tumors harbored an increased presence of surface SSEA3, SSEA4, TROP2, and Gb3 when compared to their non-tumor ER-positive counterparts (Fig. 5B).

In conclusion, epithelial and stromal-like cells detected in BCa tumors are positive for native GSL-related epitopes, which are heterogeneously distributed. SSEA1 is present preferentially on the surface of epithelial cells, while SSEA3, SSEA4, GD2, and Gb3 express a high degree of heterogeneity dependent on ER status and/or the type of breast tissue.

## Discussion

The content and distribution of glycosphingolipids in normal mammary tissue are generally assumed to be present in negligible amounts, however, contradictory reports exist. Aberrant over-representation and *de novo* synthesis of specific GSL species stand as prominent features of solid tumors (1). Most studies describe the expression pattern of GSL species based on the immunoreactivity of histological specimens with specific antibodies; only one study published by Marquina *et al.* directly analyzed the content of sialylated GSLs by gas chromatography combined with mass spectrometry and by quantitative thin-layer chromatography (5). In the context of the major cellular types present in mammary tissue such as luminal cells, basal cells, and resident macrophages, there is no relevant information about their GSL composition. Due to the intracellular origin of milk fat globules, the concentration of specific GSLs in human milk could indicate the presence of corresponding GSLs in mammary epithelial cells; however, even if so, such assumptions can be taken into account only during the lactation period and without deeper knowledge of cell surface detail (42, 43). The lack of systematic studies mapping the content of GSLs in the native microenvironment of mammary tissue is apparent and originates from restricted access to fresh, healthy mammary tissue samples and limited methodological possibilities for single-cell-based GSL detection (44). Immunohistochemistry of GSL-related epitopes is limited by at least three facts - antibodies detecting GSLs usually recognize native epitopes, and the compatibility of antibodies with fixatives is questionable. Furthermore, alcohol solutions used during standard tissue processing release glycosphingolipids into the solvent phase (similarly as in the HPLC-MS/MS procedure), thus damaging to analyzed epitopes on the cell surface. Moreover, the SSEA1 antibody was reported in neural stem cells to detect glycoproteins as the immunoreactivity of this epitope was stably present in the cells even after their exposure to GSL-dissolving methanol (45). Defining all these obstacles and after thorough in-house validation of commercial antibodies recognizing GSL-related epitopes, we established a multicolor one-tube protocol for the single-cell surface detection of GSLs under native conditions using spectral flow cytometry. Together with lineage-specific and EMT-related surface markers, we analyzed *in vitro* models of breast cells and clinical samples of non-tumor and tumor breast tissue to draw the map of the native surface distribution of GSL-related epitopes in the mammary tissue microenvironment. We were not aware of the fact that selected commercial antibodies detecting GSLs could generate non-specific signals when used for native surface staining, however, we could not ignore this possibility. Therefore, GSL antibodies were all validated and titrated in various cell models previously reported as positive or negative for particular GSL species. We compared flow cytometry data with the results of HPLC-MS/MS; we used the siRNA-based knockdown of enzymes involved in GD2 or SSEA3 biosynthesis to reduce their surface presence, and we detected a loss of fluorescent signals generated by primary conjugates in the presence of methanol (data not shown). Although these validations provided results supporting the specificity of tested antibodies, we cannot exclude the possibility of non-specific binding to the epitope related or similar to the sugar moiety used as the immunogen. Even with this handicap, we believe that surface GSLs have much to offer in the single-cell profiling of the tumor microenvironment and may contribute to the deconvolution of tumor heterogeneity and plasticity.

This study aims to answer several questions. First, we determined whether glycosphingolipids may serve as surface tags for the categorization of phenotypic plasticity in breast tissue, namely epithelial-mesenchymal plasticity. Results from metabolic profiling of GSLs in epithelial HMLE and mesenchymal HMLE-EMT breast cells showed the enrichment of epithelial cells for the Gb3 globoside and the enrichment of mesenchymal cells for the gangliosides GD3 and GD2. These findings are in accordance with a recently published study showing the same phenotype-dependent pattern of GSL distribution in ovarian cancer cells (28). Authors have described the enrichment of globosides in cancer cells characterized by E-cadherin expression, while elevated levels of genes encoding the biosynthesis of gangliosides were observed in cells expressing mesenchymal markers, namely vimentin.

Our observation of enhanced *ST8SIA1* gene expression in cells with the mesenchymal phenotype correlates with the observations showing a positive association of *ST8SIA1* expression with mesenchymal status and with the role of *ST8SIA1* in the regulation of the contact inhibition and maintenance of the mesenchymal phenotype (28, 46, 47). In breast cancer, *ST8SIA1* regulates the EMT process, tumor growth, and metastasis (47, 48). Moreover, in a recent study, authors proposed that GSL composition on the surface of ovarian cancer cells determines the EMT state (28). Our results from the analyses of GSL surface profiles confirm the presence of the GD2 positive subpopulation in cell lines with the mesenchymal phenotype (HMLE-EMT, MCF10A LXSN V12), however, breast cancer epithelial cells either of *in vitro* (T47D, SKBR3, MCF7) or clinical origin (EpCAM+/CD31-/CD45-/CD90-) were positive for GD2 as well. Similarly, although metabolic profiling revealed the strong enrichment of cells with epithelial phenotype for the Gb3 species, we did not observe increased Gb3 surface positivity in epithelial cells compared with mesenchymal cells either in *in vitro* or clinical samples. This observation can be partly explained by the fact that mesenchymal cells actively promote the synthesis of gangliosides rather than directly reduce the synthesis of the GSLs of the globo- or lacto-series; they harbor considerable levels of Gb3 as detected by HPLC-MS/MS (BT549 cells, data not shown), however, the localization of Gb3 into the cell surface is a standalone process regulated separately. This hypothesis is supported by our observation that Gb3 species are present in detectable levels in mesenchymal cells as well. Additionally, the mRNA levels of the *A4GALT* gene, which governs their synthesis, remain unchanged. Meanwhile, the expression levels of genes responsible for the synthesis of gangliosides (*ST3GAL5*, *B4GALNT1*, *ST8SIA1*, *ST8SIA5*) are significantly increased. The shift of glycolipid synthesis from one series to another during oncogenic transformation has been documented (1). In particular, the pro-ganglioside metabolic shift of mesenchymal cells is apparent mainly in cell line orthologs represented by, for example, epithelial HMLE and mesenchymal HMLE-EMT cells or between clones derived from a common parental cell line (e.g. the MCF10A LXSN model, CRISPR clones reported by Cumin *et al.*) (28) or during differentiation of neural cells from a common stem cell ancestor (49). However, its extrapolation to the general scale that would allow surface Gb3/GD2-dependent stratification of EMT state is, without the cell origin context, challenging and less reliable.

Gangliosides enriched in mesenchymal-like cells are acidic GSL species, with the basic structure of lactosylceramide undergoing serial steps of mono-, di-, or tri-sialylation, which is the incorporation of the sialic acid molecule into their sugar chains (49). Meanwhile, GSLs of the Globo-series such as SSEA4 or protein markers of epithelial cells such as EpCAM are sialylated as well, thus pointing out the necessity to evaluate the role of sialylation in the EMT process more precisely in terms of, for example, the donor molecule (N-acetylneuraminic acid or N-glycolylneuraminic acid), the character of sialylation (e.g., alpha2,6 or alpha2,8), the molecular character of the acceptor (lipid or protein), or cellular type (epithelial cancer cells or immune cells) (49, 50). Generally, aberrant sialylation present on the surface of cancer cells and related immune cells has been reported in association with breast cancer progression, metastatic dissemination, and chemoresistance (51, 52, 53). During EMT, surface sialoform undergoes dynamic alterations and becomes upregulated in mesenchymal cells together with the genes driving sialic acid biosynthesis (54). In particular, sialyltransferase inhibitors suppressed breast cancer metastasis; meanwhile, a therapeutical antibody targeting GD2, dinutuximab, which is approved for the treatment of aggressive neuroblastoma, has been speculated to be favorable for TNBC therapy (41, 55, 56, 57). Our data from the siRNA-mediated knockdown of ST8SIA1 in HMLE-EMT cells shows a negative impact of GD3S enzymatic activity on the expression of epithelial markers TROP2 and CD9 (data not shown) and thus suggest the regulatory role of sialylation in the mechanisms balancing the EMT process. Considering the role of surface sialylation in the maturation of the immune system and the mediation of the interaction between cancer and immune cells, it is tempting to speculate how the surface profiling of sialylated GSLs, e.g. GD2 or SSEA4, may contribute to a better BCa diagnosis and the earlier suggestion of combined immune therapy (58, 59). Moreover, because GSLs form carbohydrate-specific intercellular junctions between adjacent cells, their presence on the surface of cells belonging to the different intratumoral compartments holds promise for future investigations focused on the disruption of the metastatic cascade through deterioration of GSL-mediated intercellular communication between cancer cells and the cellular components of circulatory systems.

The second aim of this study was to answer the question of whether the profiling of surface GSLs present in the microenvironment of non-tumor and breast tumor tissue may serve as a tool for the description of intratumoral heterogeneity. If yes, is it possible to identify surface pairs of protein and/or lipid molecules that would be simultaneously present in specific cell subpopulations? We identified the enrichment of epithelial EpCAM+ (CD31-/CD45-/CD90-) breast cells for surface GD2, SSEA3, and the hybrid lipid/protein epitope SSEA1. Interestingly, besides epithelial-like cells, GD2, SSEA3, and TROP2 were also detected on the surface of CD90+ (CD31-/CD45-/EpCAM-) stromal-like cells present in the TNBC microenvironment and in dependence on ER status. Such information is valuable in terms of future perspectives as it suggests co-expressed targets present exclusively on the surface of cancer cell subpopulations specific for clinically challenging subtypes of BCa. In conclusion, the profiling of surface GSL-related epitopes together with specific protein molecules represented by markers of phenotypic plasticity (e.g., EpCAM) or by approved clinical targets (e.g., TROP2) (60, 61) is a promising strategy for the future expansion of therapeutic possibilities namely in clinically aggressive breast cancer subtypes such as TNBC.

## Data availability

The datasets generated during and/or analyzed during the current study are available from the corresponding authors on reasonable request.

### Supplemental data

This article contains supplementary material (supplemental Figs. S1–S7 and supplemental Tables S1–S4).

### Ethics statement

All human tissue samples were obtained at Masaryk Memorial Cancer Institute (MMCI), Brno, Czech Republic, based on approval of the Ethical Committee of the MMCI (Ref. No. 2017/1894/MOU).

### Informed consent

Informed consent was obtained from all individual participants included in the study.

## Conflict of interest

The authors declare that they have no conflicts of interest with the contents of this article.
