# Supplementary material for: Single-cell profiling of surface glycosphingolipids opens a new dimension for deconvolution of breast cancer intratumoral heterogeneity and phenotypic plasticity
Source: J Lipid Res. 2024 Jul 30;65(9):100609. doi: 10.1016/j.jlr.2024.100609 (PMC11405820; doi:10.1016/j.jlr.2024.100609)
Supplement: Supplementary data [file mmc1.docx]

**Single-cell profiling of surface glycosphingolipids opens a new dimension for deconvolution of breast cancer intratumoral heterogeneity and phenotypic plasticity**

Jiřina Procházková^1#^, Radek Fedr^1,2^, Barbora Hradilová^1,3^, Barbora Kvokačková^1,2,3^, Josef Slavík^4^, Miroslav Machala^4^, Ondrej Kováč^4^, Pavel Fabian^5,6^, Jiří Navrátil^6^, Simona Kráčalíková^7^, Monika Levková^7^, Petra Ovesná^8^, Jan Bouchal^7,9^ and Karel Souček^1,2,3#^

**Supplemental data**

Figures 1-7

Tables 1-4


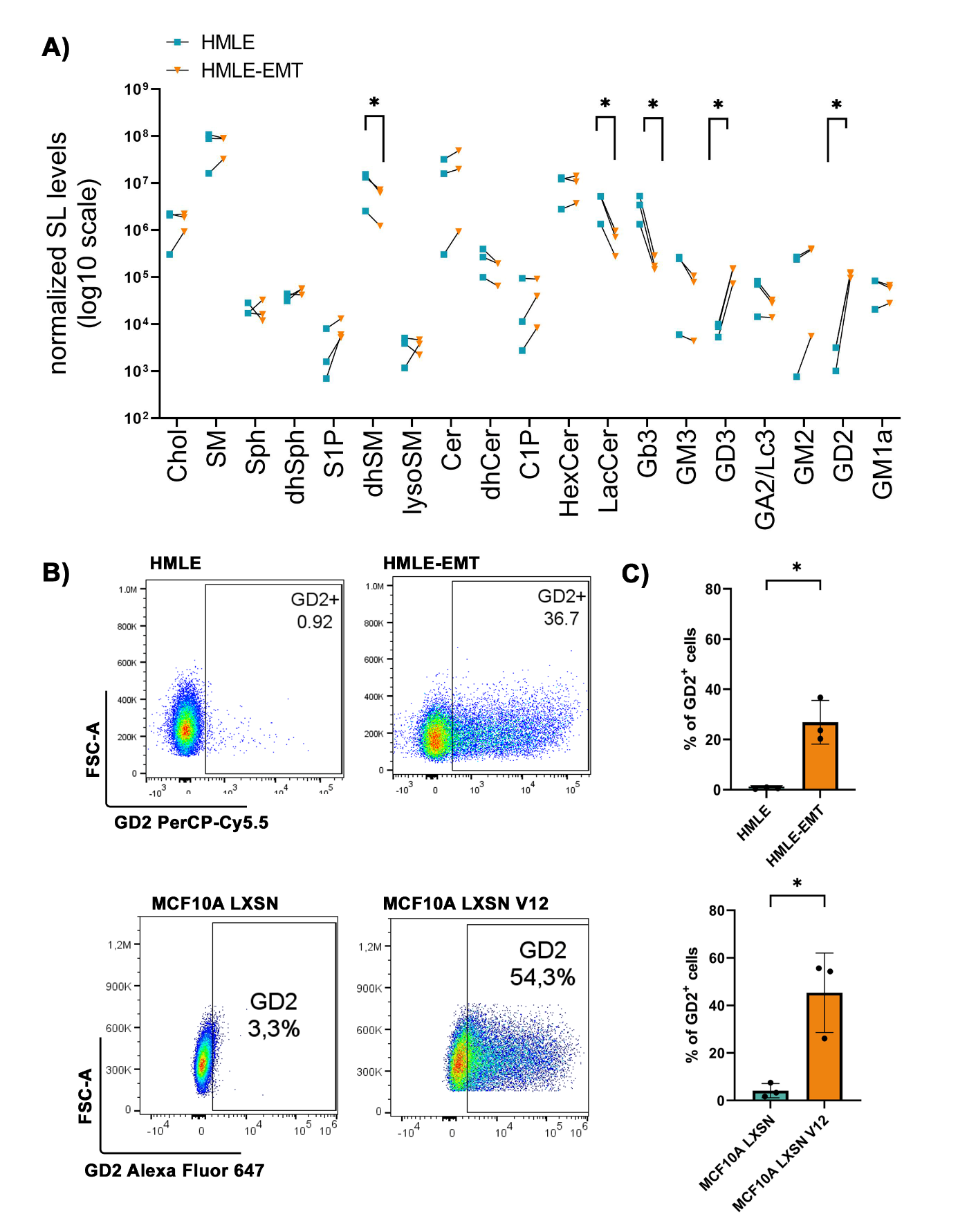


**Supplemental Figure 1. Analysis of sphingolipidome in *in vitro* models of breast EMT.** Epithelial (turquoise) HMLE and mesenchymal (orange) HMLE-EMT cells were analyzed for the levels of sphingolipids and glycosphingolipids by HPLC-MS/MS. Significantly deregulated lipid species are marked with asterisks (n= 3, *p < 0.01, paired t-test). B) Representative dot plots showing GD2- and GD2+ cells as analyzed by conventional flow cytometry. C) Average percentage of GD2+ and GD2- cells in epithelial (HMLE, MCF10A LXSN) and mesenchymal (HMLE-EMT, MCF10A LXSN V12) in vitro models. Differences between EMT phenotypes were evaluated by unpaired t-test with * p < 0.05, n = 3.


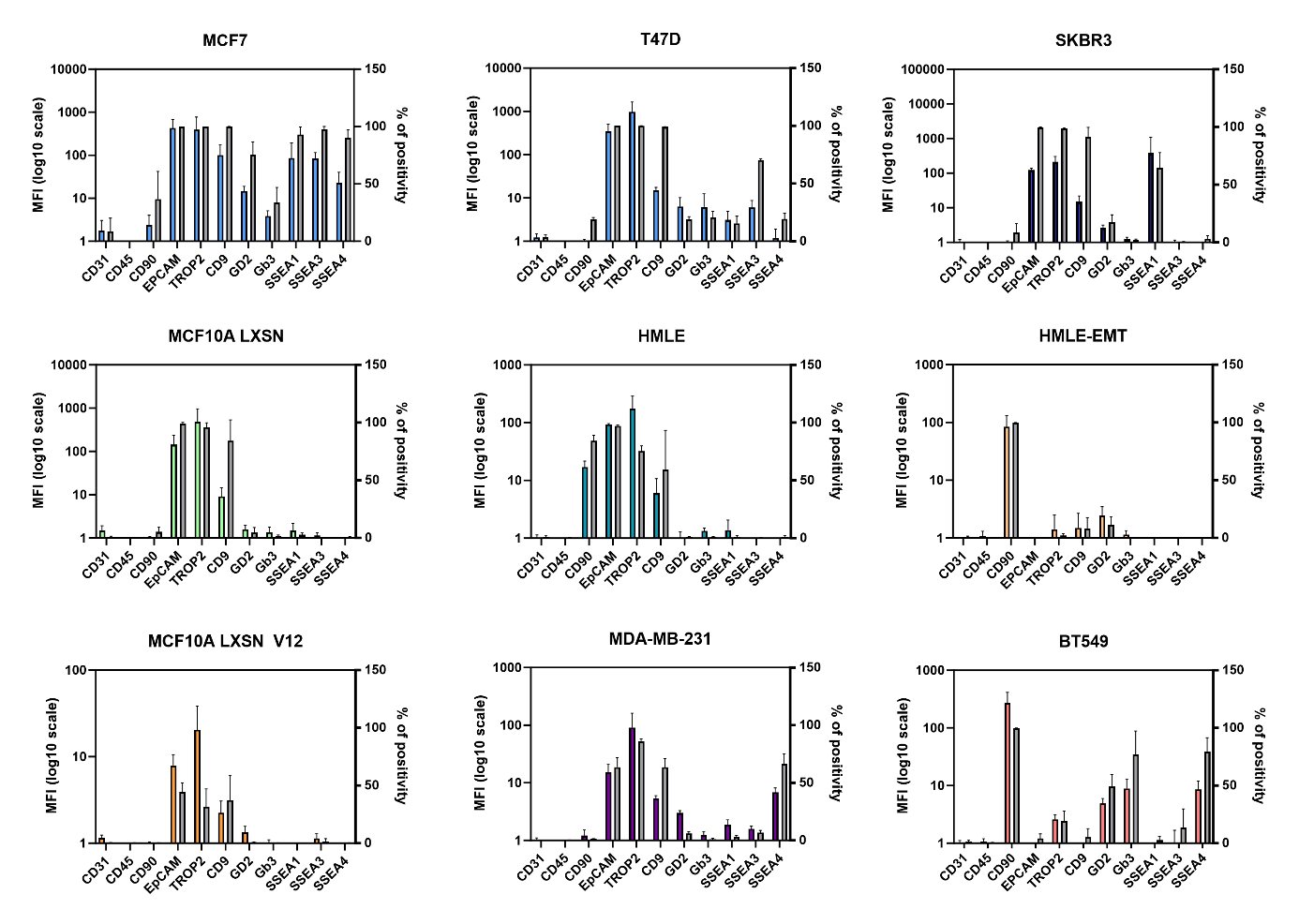


**Supplemental Figure 2. Surface profiling of breast epithelial and mesenchymal cell lines.** Breast cell lines were stained with a multi-color panel of antibodies specified in Supplementary Table 3, surface presence of GSL-related epitopes, lineage-specific markers, and EMT-related markers was profiled. The percentage of positivity (grey bars) and median fluorescence indexes (MFI; colored bars) for each epitope are summarized (n= 3).


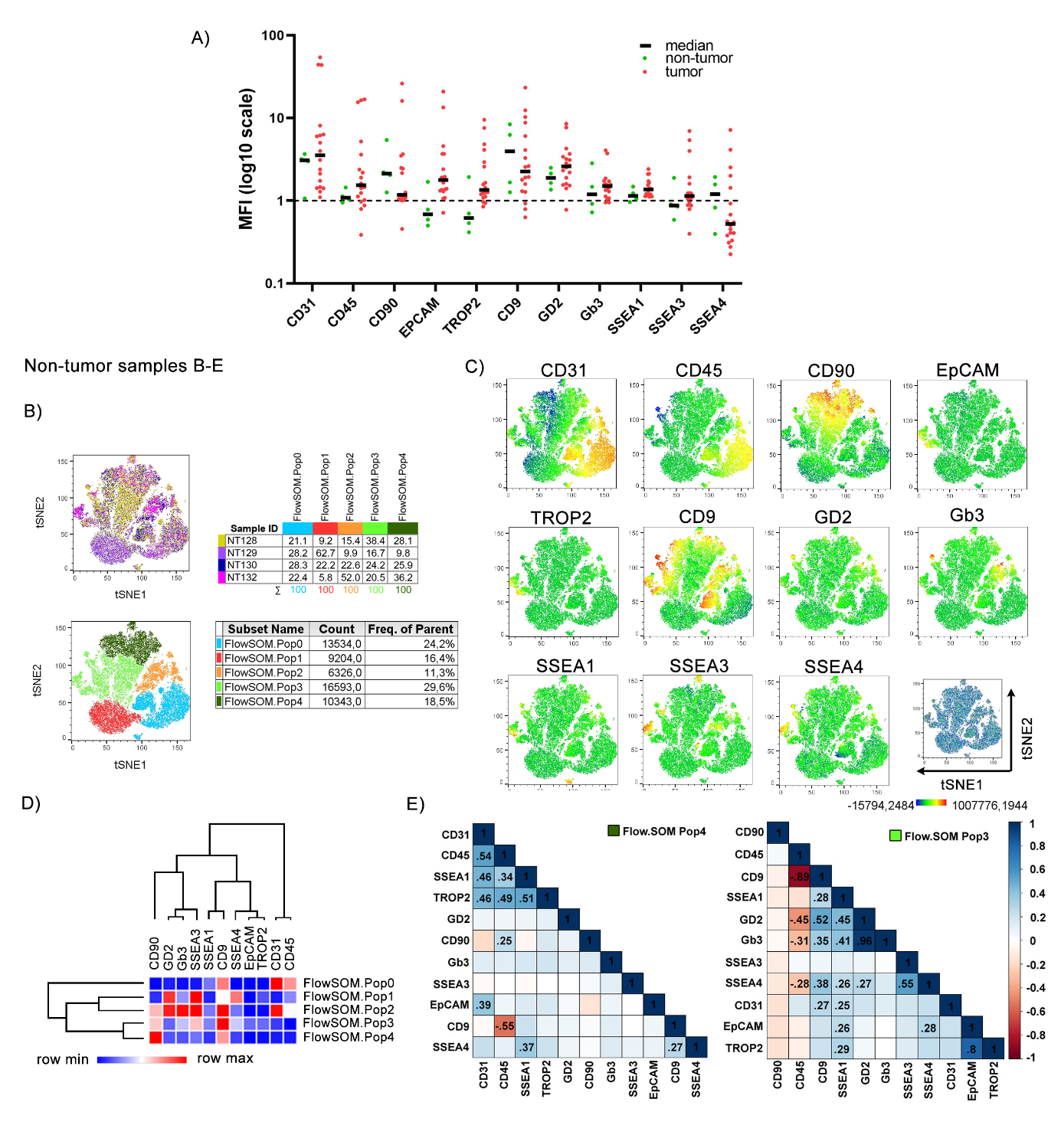


**Supplemental Figure 3. Characterization of breast tissue heterogeneity by surface profiling of GSL- and EMT-related epitopes in non-tumor clinical samples.** A) Individual MFI values of each epitope analyzed in all non-tumor and tumor samples are visualized together with the calculated median. B) tSNE plots showing pooled cells from 4 non-tumor samples colored by sample ID (up) or by FlowSOM clusters (down). Tables show the percentual distribution of FlowSOM clusters in each sample and their frequency in the total pool of cells. C) tSNE plots colored by expression of surface epitopes. The color corresponds to the fluorescence intensity (red - high; blue - low). D) Heatmap depicts hierarchical clustering and expression intensity of surface markers in identified FlowSOM clusters. E) FlowSOM cluster-specific correlation matrices of analyzed epitopes as calculated from single-cell fluorescence intensities. Only statistically significant correlation coefficients r > 0.25 (blue) or r < -0.25 (red) are shown.


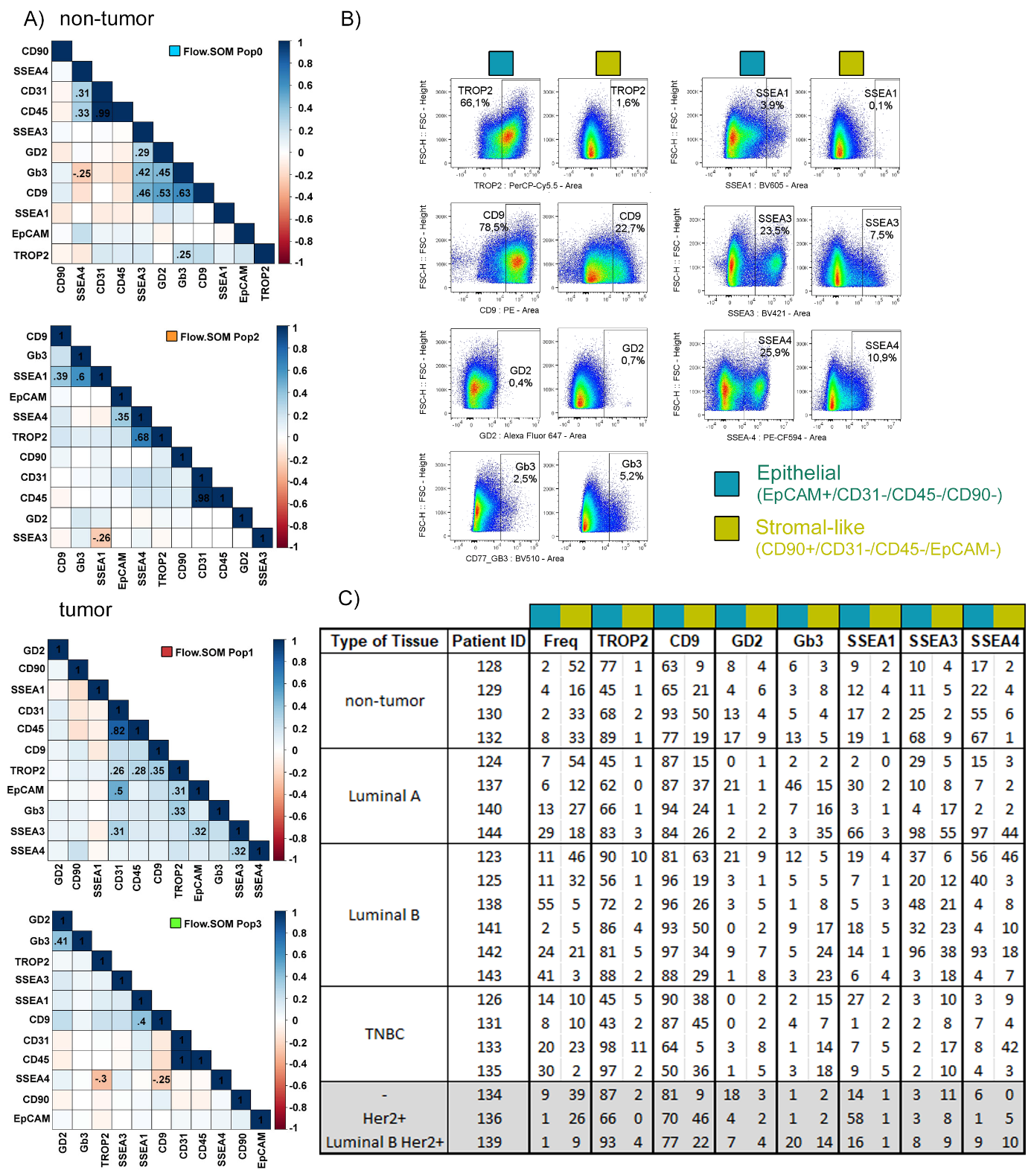


**Supplemental Figure 4. Surface profiling of cellular clusters identified in clinical samples.** A) FlowSOM cluster-specific correlation matrices of analyzed epitopes in non-tumor and tumor samples as calculated from single-cell fluorescence intensities. Only statistically significant correlation coefficients r > 0.25 or r < -0.25 are shown. Selected clusters are enriched for hematopoietic and endothelial cells present in breast tissue microenvironment. B) Dot plots of pooled epithelial (blue square) or stromal-like (yellow square) cells identified in tumor samples show a percentage of cellular positivity for specific epitopes. C) % of positivity for each marker in epithelial and stromal-like cells as identified in each patient. The patient cohort is divided based on the PAM50 classification.


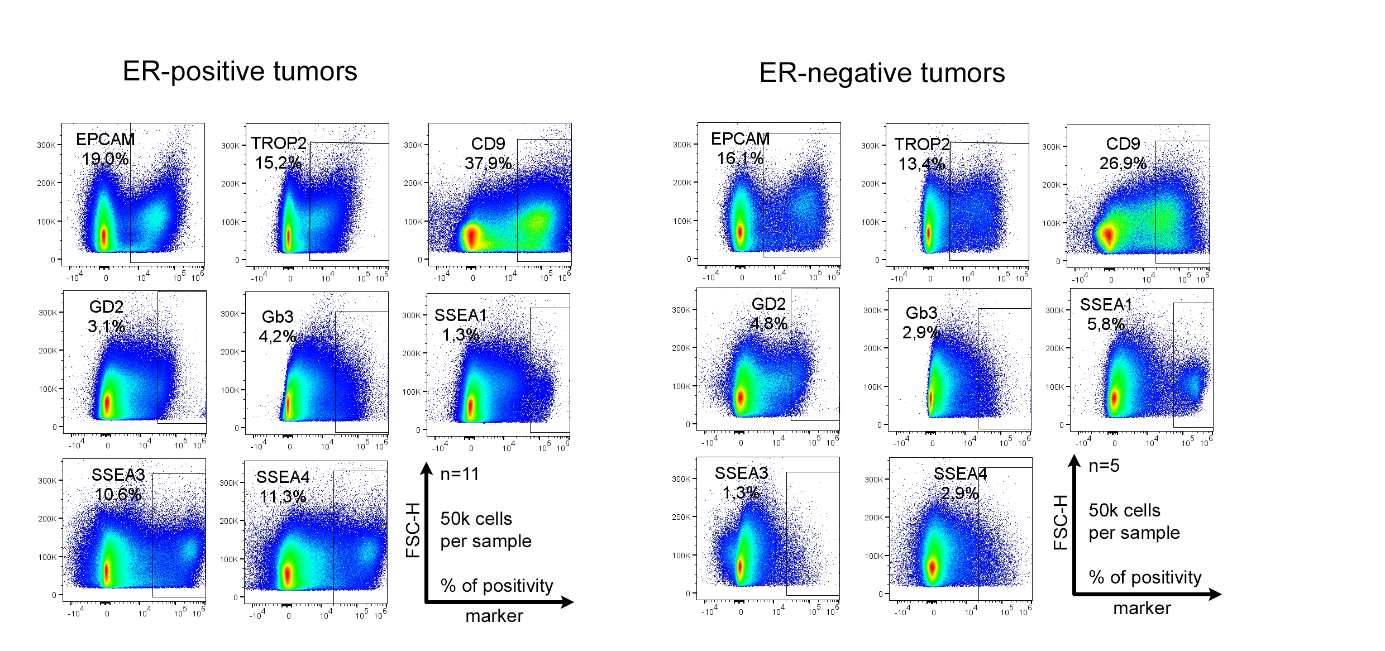


**Supplemental Figure 5. Impact of estrogen receptor (ER) status on surface expression of GSLs and EMT markers.** 50k of viable single cells per sample were concatenated without further lineage selection to visualize % of positivity for analyzed surface molecules. Dot plots of pooled cells from eleven ER-positive and five ER-negative tumors show the percentage of cellular positivity for specific epitopes and indicate differences in surface expression of SSEAs between compared groups.


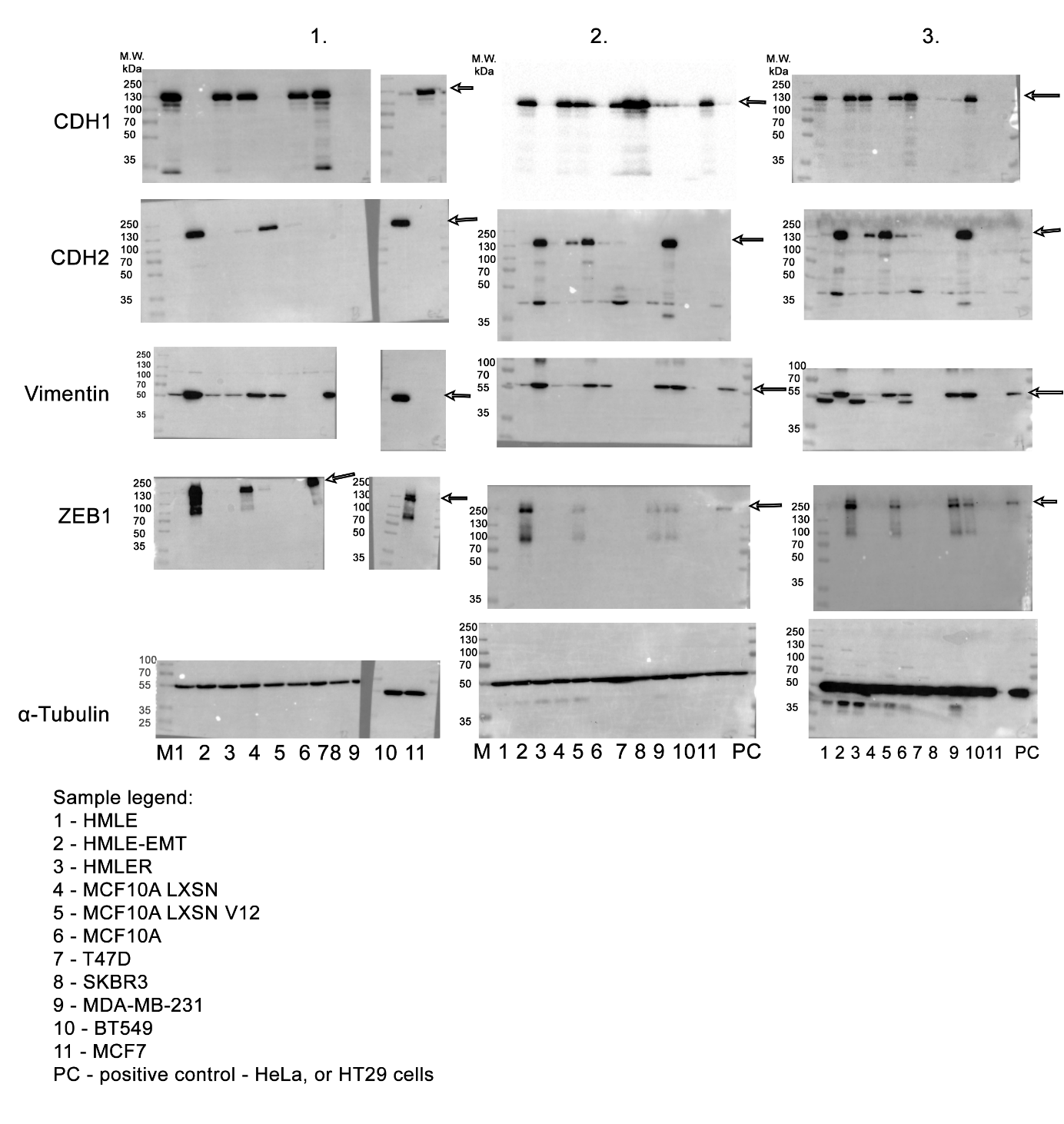


**Supplemental Figure 6. Overview of uncropped western blots from three independent repetitions.** Arrows indicate specific bands for each protein, and molecular weight (M.W.) is depicted in kilodaltons and determined based on a pre-stained protein standard ladder. PC stands for positive control.


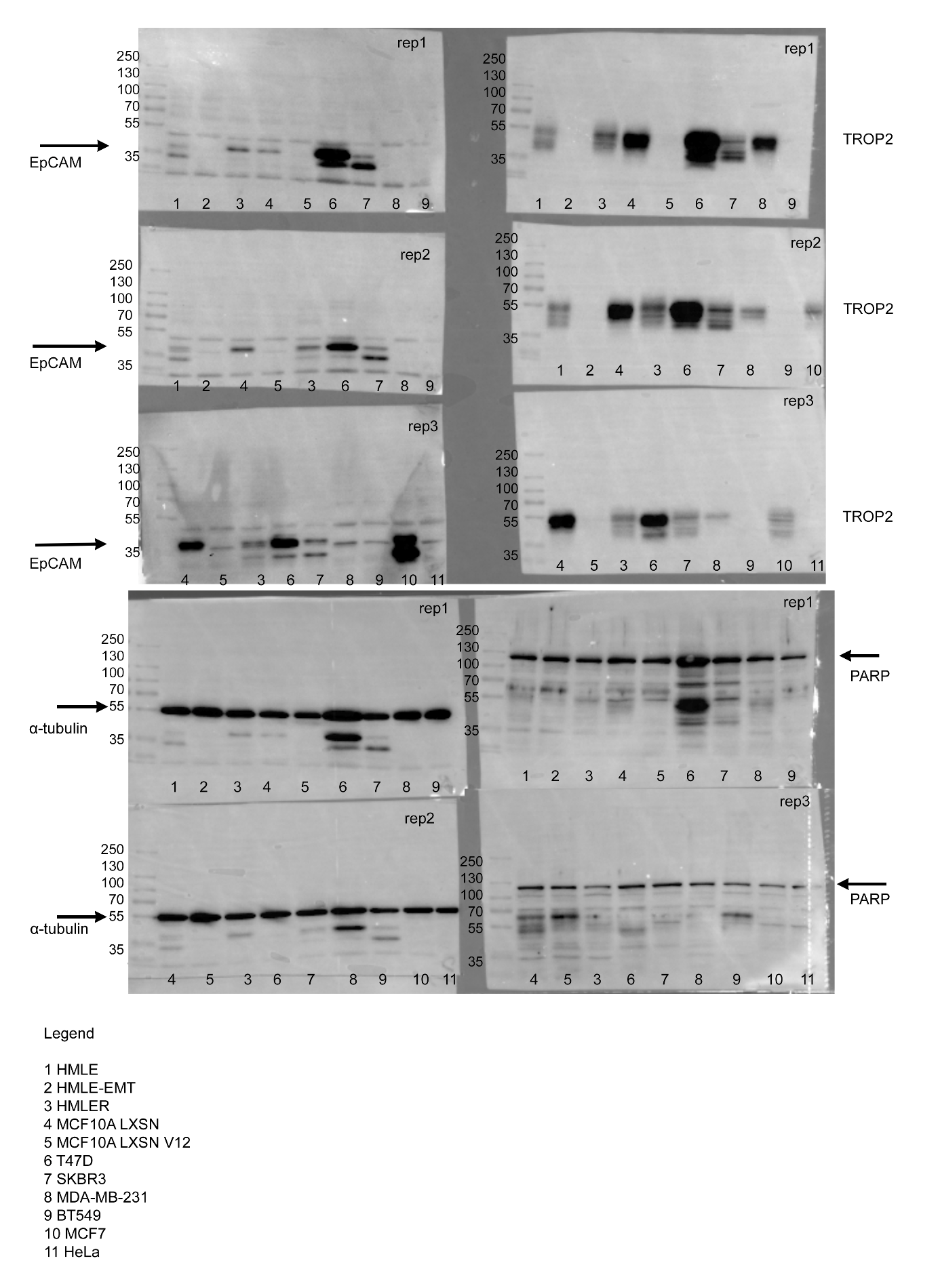
**Supplemental Figure 7. Overview of uncropped western blots detecting EpCAM and TROP2 from three independent repetitions.** Arrows indicate specific bands for each protein, and molecular weight (M.W.) is depicted in kilodaltons and determined based on a pre-stained protein standard ladder.

**Supplemental Tables 1-4:**

**Supplemental Table 1.** Total normalized levels of sphingolipid species analyzed by HPLC-MS/MS in HMLE and HMLE-EMT cells (an extra .xlsx file).

| **Gene / Protein** | **Cat.no.** | **Assay no. / Species** | **Provider** |
| --- | --- | --- | --- |
| B4GALT5 | 4448892 | Hs00941041_m1 | ThermoFisher (TFS) |
| B4GALT6 | 4448892 | Hs00999574_m1 | TFS |
| B3GNT5 | 4448892 | Hs00908059 | TFS |
| A4GALT | 4448892 | Hs00213723_m1 | TFS |
| ST3GAL5 | 4448892 | Hs01105377_m1 | TFS |
| NEU3 | 4448892 | Hs00198406_m1 | TFS |
| B4GALNT1 | 4351372 | Hs01110791_g1 | TFS |
| B3GALT4 | 4448892 | Hs00534104_s1 | TFS |
| ST8SIA1 | 4448892 | Hs01124292_m1 | TFS |
| ST8SIA5 | 4448892 | Hs00203298_m1 | TFS |
| HEXA | 4448892 | Hs00942655_m1 | TFS |
| HEXB | 4448892 | Hs01077594_m1 | TFS |
| GAPDH | 4331182 | Hs02786624_g1 | TFS |
| CDH1 | 610182 | mouse | BD Pharmingen |
| CDH2 | 610920 | mouse | BD Pharmingen |
| Vimentin | SAB4300676 | rabbit | Sigma-Aldrich / MERCK |
| ZEB1 | 70512 | rabbit | Cell Signaling |
| alpha-tubulin | T9026 | mouse | Sigma-Aldrich / MERCK |
| EpCAM | sc-25308 | mouse | Santa Cruz |
| TROP2 | 11-898-C100 | mouse | Exbio |
| PARP-1 | Sc-7150 | rabbit | Santa Cruz |

**Supplemental Table 2.** Taqman assays and western blotting antibodies used in this study.

| **Viability/Antibody/Isotype** | **Conjugate** | **Dilution** | **Cat. no.** | **Manufacturer** |
| --- | --- | --- | --- | --- |
| ZOMBIE NIR | NIR | 1:500 | 423105 | Biolegend |
| CD31 | BV786 | 1:320 | 744757 | BD Horizon |
| CD45 | FITC | 1:80 | 2120030 | SONY |
| CD90 | AF700 | 1:20 | 2240600 | SONY |
| EPCAM | PE-Cy7 | 1:80 | 324222 | Biolegend |
| Gb3/CD77 | BV510 | 1:20 | 563630 | BD Horizon |
| GD2 | AF647 | 1:80 | 357318 | Biolegend |
| CD9 | PE | 1:10 | 1P-208-T100 | EXBIO |
| SSEA-1 | BV605 | 1:20 | 2215155 | SONY |
| SSEA-3 | BV421 | 1:80 | 562706 | BD Horizon |
| SSEA-4 | PE-CF594 | 1:20 | 562487 | BD Horizon |
| TROP2 | PerCP-Cy5.5 | 1:80 | T9-898-T100 | Exbio |
| mouse IgG1 | BV786 | 1:320 | 744757 | BD Horizon |
| mouse IgG1 | FITC | 1:80 | 2120030 | SONY |
| mouse IgG1 | AF700 | 1:20 | 2240600 | SONY |
| mouse IgG2b | PE-Cy7 | 1:80 | 324222 | Biolegend |
| mouse IgM | BV510 | 1:20 | 563630 | BD Horizon |
| mouse IgG2a | AF647 | 1:80 | 357318 | Biolegend |
| mouse IgG1 | PE | 1:10 | 1P-632-C100 | Exbio |
| mouse IgG1 | BV605 | 1:20 | 2215155 | SONY |
| rat IgM | BV421 | 1:80 | 562706 | SONY |
| mouse IgG3 | PE-CF594 | 1:20 | 532487 | BD Horizon |
| mouse IgG2b | PerCP-Cy5.5 | 1:80 | T9-898-T100 | Exbio |
| Live/Dead Yellow | 405/570 | 1:500 | L34959 | ThermoFisher |
| CD9 | CF-Blue | 1:20 | 9CFB-100T | Immunostep |
| CD326/ EpCAM | PE-Dazzle594 | 1:200 | 2221160 | SONY |
| CD29/ITGB1 | SB600 | 1:20 | 63-0299-42 | ThermoFisher |
| CD49c/ ITGA3 | BV711 | 1:400 | 744520 | BD Biosciences |
| TROP2 | APC | 1:20 | FAB650A | R&D |
| GD2 | PerCP-Cy5.5 | 1:20 | 357312 | BioLegend |
| mouse IgG2a | CF-Blue | 1:20 | ICIGG2ACFB-100 | Immunostep |
| mouse IgG2b | PE-Dazzle594 | 1:200 | 2601790 | SONY |
| mouse IgG1 | SB600 | 1:20 | 63-4714-82 | ThermoFisher |
| mouse IgG1 | BV711 | 1:400 | 563044 | BD Biosciences |
| mouse IgG2a | APC | 1:20 | IC003A | R&D |
| rat IgG2a | PerCP-Cy5.5 | 1:40 | 400532 | BioLegend |

**Supplemental Table 3.** Antibodies and isotype controls used in multi-color flow cytometric assays

| **Patient ID**​ | **Age** | **Sample ID**​ | **tissue type**​ | **BCa subtype**​ | **histology**​ | **grade**​ | **pT**​ | **pN**​ | **L**​ | **V**​ | **M**​ | **ER (%)**​ | **PR (%)**​ | **ki-67 (%)**​ | **HER2**​ |
| --- | --- | --- | --- | --- | --- | --- | --- | --- | --- | --- | --- | --- | --- | --- | --- |
| 1​ | 47 | BCa123​ | tumor​ | luminal B​ | NST​ | 2​ | 2​ | 0​ | 0​ | 0​ | 0​ | 100​ | 95​ | 31​ | 0​ |
| 2​ | 67 | BCa124​ | tumor​ | luminal A​ | NST​ | 1​ | 2​ | 0​ | 0​ | 0​ | 0​ | 90​ | 0​ | 21​ | 0​ |
| 3​ | 50 | BCa125​ | tumor​ | luminal B​ | NST​ | 2​ | 1c​ | 2a​ | 1​ | 0​ | 0​ | 100​ | 0​ | 31​ | 0​ |
| 4​ | 58 | BCa126​ | tumor​ | TNBC​ | NST​ | 3​ | 1c​ | 0​ | 0​ | 0​ | 0​ | 1​ | 0​ | 53​ | 0​ |
| ​ |  | BCa127​ | tumor​ | ​ | ​ | ​ | ​ | ​ | ​ | ​ | ​ | ​ | ​ | ​ | ​ |
| 5​ | 45 | NT128​ | non-tumor​ | luminal B​ | NST​ | 3​ | 1b​ | 1a​ | 0​ | 0​ | 0​ | 100​ | 0​ | 21​ | 0​ |
| ​ |  | NT129​ | non-tumor​ | ​ | ​ | ​ | ​ | ​ | ​ | ​ | ​ | ​ | ​ | ​ | ​ |
| 6​ | 81 | NT130​ | non-tumor​ | TNBC​ | NST​ | 3​ | 2​ | 1a​ | 0​ | 0​ | 0​ | 0​ | 0​ | 61​ | 0​ |
| ​ |  | BCa131​ | tumor​ | ​ | ​ | ​ | ​ | ​ | ​ | ​ | ​ | ​ | ​ | ​ | ​ |
| 7​ | 71 | NT132​ | non-tumor​ | TNBC​ | NST​ | 3​ | 2​ | 0​ | 0​ | 0​ | 0​ | 0​ | 0​ | 83​ | 0​ |
| ​ |  | BCa133​ | tumor​ | ​ | ​ | ​ | ​ | ​ | ​ | ​ | ​ | ​ | ​ | ​ | ​ |
| 8​ | 40 | BCa134​ | tumor​ | -​ | pTisL​ | ​ | pTisL​ | 0​ | ​ | ​ | 0​ | ​ | ​ | ​ | 0​ |
| 9​ | 67 | BCa135​ | tumor​ | TNBC​ | NST​ | 3​ | 3​ | 3a​ | 1​ | 1​ | 0​ | 0​ | 0​ | 73​ | 0​ |
| 10​ | 34 | BCa136​ | tumor​ | Her2+​ | NST​ | 3​ | 1c​ | 0​ | 0​ | 0​ | 0​ | 0​ | 0​ | 45​ | 1​ |
| 11​ | 85 | BCa137​ | tumor​ | luminal A​ | NST​ | 2​ | 2​ | 0​ | 0​ | 0​ | 0​ | 100​ | 100​ | 14​ | 0​ |
| 12​ | 71 | BCa138​ | tumor​ | luminal B​ | invasive lobular​ | 2​ | 3​ | 3a​ | 0​ | 0​ | 0​ | 100​ | 100​ | 16​ | 0​ |
| 13​ | 48 | BCa139​ | tumor​ | luminal B Her2+​ | NST​ | 3​ | 2​ | 0​ | 0​ | 0​ | 0​ | 100​ | 90​ | 59​ | 1​ |
| 14​ | 46 | BCa140​ | tumor​ | luminal A​ | NST​ | 2​ | 2​ | 1a​ | 0​ | 0​ | 0​ | 100​ | 100​ | 23​ | 0​ |
| 15​ | 60 | BCa141​ | tumor​ | luminal B​ | NST​ | 2​ | 4b​ | 2a​ | 0​ | 0​ | 0​ | 100​ | 100​ | 56​ | 0​ |
| 16​ | 71 | BCa142​ | tumor​ | luminal B​ | NST with mucinous component​ | 2​ | 2​ | 0​ | 0​ | 0​ | 0​ | 100​ | 20​ | 47​ | 0​ |
| 17​ | 72 | BCa143​ | tumor​ | luminal B​ | NST​ | 3​ | 2​ | 0​ | 0​ | 0​ | 0​ | 100​ | 5​ | 43​ | 0​ |
| 18​ | 67 | BCa144​ | tumor​ | luminal A​ | invasive lobular carcinoma​ | 2​ | 2​ | 0​ | 0​ | 0​ | 0​ | 100​ | 70​ | 18​ | 0​ |
| NST - non-special type; pTisL - tumor in situ only | | | | | | | | | | | | | | | |

**Supplemental Table 4.** Patient cohort description and sample identification.
